# Long-term exposure to air pollution and metabolites in children and young adults in a Swedish birth cohort

**DOI:** 10.1038/s41370-025-00810-1

**Published:** 2025-10-03

**Authors:** Shizhen He, Baninia Habchi, Romanas Chaleckis, Natalia Hernandez-Pacheco, Anna Bergström, Anne-Sophie Merritt, Inger Kull, Kristina Eneroth, Matteo Bottai, Göran Pershagen, Simon Kebede Merid, Sophia Björkander, Zhebin Yu, Erik Melén, Olena Gruzieva, Craig E. Wheelock, Susanna Klevebro

**Affiliations:** 1 Institute of Environmental Medicine, Karolinska Institute, Stockholm, Sweden; 2 Unit of Integrative Metabolomics, Institute of Environmental Medicine, Karolinska Institute, Stockholm, Sweden; 3 Department of Occupational and Environmental Health, Nagoya City University Graduate School of Medical Sciences, Nagoya, Japan; 4 Department of Clinical Science and Education, Karolinska Institutet, Södersjukhuset, Stockholm, Sweden; 5 Centre for Occupational and Environmental Medicine, Region Stockholm, Stockholm, Sweden; 6 Sachs’ Children and Youth Hospital, Södersjukhuset, Stockholm, Sweden; 7 Environment and Health Administration, SLB-analys, Stockholm, Sweden; 8 Division of Biostatistics, Institute of Environmental Medicine, Karolinska Institute, Stockholm, Sweden; 9 Department of Respiratory Medicine and Allergy, Karolinska University Hospital, Stockholm, Sweden

**Keywords:** air pollution, children, particulate matter, urine metabolomics, young adults

## Abstract

**Background:**

The biochemical dysregulation underlying the adverse health effects of exposure to air pollution (AP) remains unclear.

**Objective:**

The objective of this study was to explore associations between long-term exposure to AP and the urinary metabolome.

**Methods:**

In the Swedish birth cohort BAMSE (*n* = 4089), urine samples were collected from a subset of participants attending clinical examination at the 4-year follow-up and from all participants attending clinical examination at the 24-year follow-up. Among paired samples and children with diagnosis of asthma and/or low lung function, non-targeted screening using liquid chromatography high-resolution mass spectrometry was applied to 4-year samples (*n* = 612) and 24-year samples (*n* = 846) and metabolites were annotated based on standard matching to in-house compound libraries (*n* = 260 metabolites). Time-weighted average exposure to air pollutants (*i.e*., particulate matter with diameter ≤10μm (PM_10_), ≤2.5 μm (PM_2.5_), and nitrogen oxides (NO_x_)) during the first year of life and the year prior to urine collection was estimated using validated dispersion models. The association between AP exposure and urine metabolites was estimated cross-sectionally using exponential regression.

**Results:**

AP exposure was overall positively associated with metabolite abundance (*p* < 0.002). However, metabolite-specific associations exhibited variability. At the 4-year follow-up, the first-year-of-life and prior-year AP exposures were positively associated with 8 purine/pyrimidine derivative metabolites (*e.g*., an increase of 2.8 μg/m^3^ (interquartile range) in PM_10_ during the first year of life was associated with a 1.21-fold increase in 1,7-dimethylxanthine, *p* = 3.87E−05). We also observed interactions between AP exposures and metabolism-related genetic variants on metabolite levels. At the 24-year follow-up, prior year AP was negatively associated with levels of six long-chain fatty acids.

**Impact:**

- Long-term exposure to air pollution alters urinary metabolites in children and young adults, revealing environmental impacts on systemic metabolism even at low levels of air pollution.

## Introduction

Air pollution is a significant public health issue worldwide and an established risk factor for morbidity and mortality [1]. Accumulating research has identified inflammation and oxidative stress to have important roles in the health effects of air pollution [2–4]. However, significant gaps remain in our understanding of the etiological mechanisms and the extent to which underlying molecular changes induced by air pollution can be detected during early childhood, particularly in the metabolome [5].

Metabolomic epidemiology has emerged as a powerful methodology to explore how air pollution exposure is affecting biological responses [6]. Metabolites are low molecular weight compounds that reflect systemic biochemistry [7, 8]. The metabolome can serve as a proxy for the internal exposome [9], which consists of the exogenous compounds entering the internal environment (*e.g*., dietary, medication-related, and ambient environmental molecules), endogenous compounds that are the products of genetic and endogenous processes stimulated by the external environment (e.g., oxidative stress, inflammation), and the interactions between exogenous and endogenous compounds. While metabolomics has been performed in many biofluids, urine offers some distinct advantages including: (1) non-invasive collection, (2) higher concentration of conjugated metabolites, (3) reflects end-state metabolism, (4) represents a wide range of biochemical processes (although it captures a narrower spectrum of biochemical processes compared to blood), and (5) provides an integrative snapshot of systemic biochemical imbalances [8, 10].

Experimental studies have found that short-term exposure to particulate air pollution can lead to metabolic dysregulation in the urine and blood metabolomes in animal models [11, 12]. Although the identified metabolic signatures vary across studies, metabolome-wide epidemiological studies in humans consistently support the role of air pollution in the perturbation of pathways related to inflammation, oxidative stress, and mitochondrial function [5, 13, 14]. The majority of studies have focused on investigating the effects of short-term air pollution exposure in adults. Data are scarce regarding the effects of air pollution on the metabolome in children [15] as well as the consequences of long-term air pollution exposure [16–20]. Furthermore, in younger populations, where genetic influences on omics profiles are generally more pronounced, the metabolism and systemic metabolome are largely shaped by both internal genetic processes and external exposures such as air pollution [21–23].

The aim of the current study was to investigate the association of long-term air pollution exposure at various life stages (i.e., during the first year of life and during the prior year to biosampling) with the internal exposome by measuring urine metabolites in children at 4 years of age and in young adults at 24 years of age, using a well-defined cohort with genetic data available. Given the potentially stronger genetic influence on metabolism in early life, we additionally explored the interaction between air pollution exposure and genetic variants in relation to the metabolism of some of the identified metabolites.

## Methods

### Study design and population

This is a cross-sectional analysis of urine metabolites at two time-points in a birth cohort with prospectively collected data. The study included participants from the population-based birth cohort BAMSE (Swedish acronym for Children, Allergy, Milieu, Stockholm, Epidemiology), consisting of 4089 infants born in four predefined areas of Stockholm, Sweden, between 1994 and 1996 [24]. Parents were invited to complete a baseline questionnaire that assessed environmental exposures, parental smoking habits, residential characteristics, lifestyle, and parental allergies. Questionnaires were answered repeatedly by the parents (up to age 16 years) and by the participants themselves (from age 12 years). For the current study, potential confounders were extracted from questionnaires from 3 months, 1 year, 4 years, and 24 years of age. Urine samples were collected at ages 4 and 24 years from 933 (23%) children and 2235 (55%) young adults, respectively. At age 4, children were assigned to urine sampling in a subgroup based on the prevalence of symptoms of allergic diseases, as described in detail previously [25]. At 24 years of age, all participants were asked to leave a urine sample at the clinical examination.

**Fig. 1:**
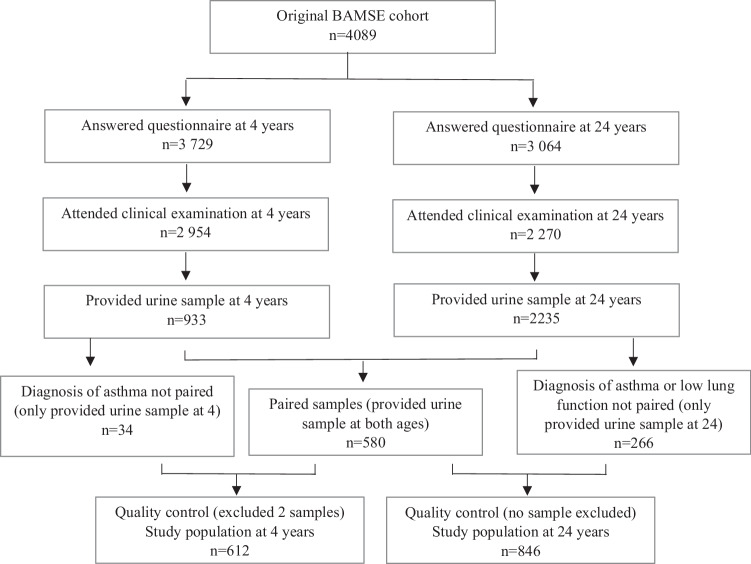
Flow chart and selection criteria for the study population.

In total, 1460 urine samples were selected for urine metabolomics analyses at 4 and 24 years of age (Fig. 1), including all participants having urine samples at both time points (580 paired samples), and additional samples from participants with a diagnosis of asthma and/or low lung function (34 additional 4-year samples plus 266 additional 24-year samples). The study population was therefore a mild asthma- and allergic symptoms-enriched population compared to the general population. Low lung function was defined as the ratio of pre-bronchodilator forced expiratory volume in one second (FEV_1_) and forced vital capacity (FVC) ratio below the lower limit of normal (LLN) based on the Global Lung Function Initiative (GLI) criteria (*i.e*., FEV_1_/FVC < LLN (GLI) at 24 years of age) [26].

### Air pollution exposure assessment

The methodology for calculating individual long-term exposure to locally emitted air pollutants has been described in detail previously [27]. In order to explore the impact of air pollution exposure during the first year of life and the year prior to biosampling, time-weighted average exposures were calculated at the individual address level for these periods. The exposures included particulate matter with a diameter ≤10 μm (PM_10_), particulate matter with a diameter ≤2.5 μm (PM_2.5_), and nitrogen oxides (NO_x_), calculated using a validated Gaussian air quality dispersion model and a wind model, both part of the Airviro Air Quality Management System (http://airviro.smhi.se). The calculations were performed on a 35-m resolution grid for addresses in the more densely populated areas of Stockholm County, such as urban areas, and a 100-m or 500-m grid in less densely populated areas. In addition, a street canyon contribution was added for addresses in the most polluted street segments in the inner city of Stockholm with multistorey houses on both sides, using the Airviro street canyon model (until 2012; www.airviro.com/airviro/ modules) and the OSPM operational street pollution model (from 2013 onwards; www.au.dk/OSPM). Emission databases were available for the years 1990, 1995, 2000, 2002, 2003, 2004, 2006, 2010, 2015, and 2020. To obtain concentrations for all years during the period of interest, the model calculations were interpolated. Annual average long-range contributions were added to the locally modeled concentrations based on continuous measurements at regional background stations.

### Outcome assessment

Urine samples were collected at clinical examination visits at the 4- and 24-year follow-ups, and stored at -80° C. A detailed description of the urine metabolomic profiling methods has been previously published [28]. Before analysis, samples were thawed and normalized based on specific gravity measurements [29]. Non-targeted screening based on liquid chromatography high-resolution mass spectrometry (LC-HRMS) was applied to acquire the metabolomic profile [28, 29]. Two independent injections were run: one in positive and one in negative acquisition mode. Quality control samples were injected after every five biological samples. ProteoWizard [30] was used for data quality check using MZmine2.53 [31]. Targeted peak detection was based on 3 technical standards (CHES, HEPES and PIPES) and 25 pre-defined metabolites [29]. The data quality was assessed based on the stability of binary pump pressures, overlaid total ion chromatograms (TICs) for QCs, extracted ion chromatograms (EICs) of lock masses for all samples and QCs only, 3D views (x = RT, y = intensity, z = m/z) of one QC sample and the reference blank sample, repeatability of the EICs of each technical standard extracted with 20 ppm tolerance and calculations of Coefficient of Variation (CVs) in the QCs (<10%) and the samples (<20%) using R scripts. Batch correction was performed using Quality Control-Robust Spline Correction [32]. The untargeted metabolomics analyses resulted in 9663 metabolite features, including 4733 features in negative ionization mode and 4930 features in positive ionization mode. The untargeted data were annotated based on an in-house library of 622 chemical standards resulting in 260 unique chemical standard-confirmed metabolites from both positive and negative ionization modes (Appendix A). Peak areas, as measures of relative abundance, were used in the statistical analyses. Extreme outliers were identified and subsequently excluded from the analysis based on the results of principal component analysis (PCA) using the 95% limit of Hotelling’s T2.

### Statistical analyses

The association analyses were conducted with the Stata (version 16.1; StatCorp) and R software (version 4.2.2). All analyses were performed cross-sectionally for 4- and 24-year samples. The main analyses were applied to annotated metabolites (level 1). Firstly, we explored the overall effect of air pollution on the urine metabolome. This metabolome-level analysis was motivated by our hypothesis that air pollution exposure may induce a systematic metabolic shift rather than isolated effects on individual metabolites in a few pathways. To investigate the association between air pollution and the urine metabolome, we initially aggregated annotated metabolites into one outcome variable (referred to as “metabolome”; details described in Appendix A). After that, a parametric model using exponential regression with “metabolite id” as a gamma-distributed random effect (to model the heterogeneity of metabolites) and an exponential distribution was performed. This analysis has the benefit of dimensionality reduction and more statistical power, but the estimate could be diluted by opposing directions of different metabolites. Missing values of metabolites were considered to imply a very low level or a biological zero. Missing values were imputed using half the minimum observed value for each metabolite, given the low levels of missingness in annotated metabolites [33].

Next, to explore specific air pollution-associated metabolites, a left-censored parametric model using exponential regression was applied for the annotated metabolites. Instead of half-minimum imputation, measurements below the minimum observed value were treated as left-censored observations [34]. The left censoring was suitable because the missing observations were not missing at random, but were rather below the detectable values available in the dataset. The impact of influential observations was assessed by winsorization, where the modeling was checked by replacing values above the 99^th^ percentile with the 99th percentile value [35]. For statistically significant associations (p < 0.05), we applied a visual bivariate assessment of the effect of influential observations via a density plot of the distribution of each metabolite over quartiles of air pollution exposure. As a complement to the main analysis, the same metabolite-specific model was applied to all-feature datasets, including non-annotated features, to perform supplementary pathway enrichment analysis.

All results are presented as fold change, comparing the predicted marginal medians when air pollution exposure was increased by one unit from the median. To enhance the comparability of the effect estimates between different exposure periods, one unit was set as one interquartile range (IQR) of air pollution levels averaged during the first year of life, corresponding to 2.8, 1.1, and 22.9 μg/m^3^ for PM_10_, PM_2.5_, and NO_x_, respectively. Multiple testing was accounted for in the metabolite-specific analyses by controlling the false discovery rate (FDR) at 5%, implementing the Benjamini-Hochberg adjustment [36]. FDR-corrected *p* < 0.05 was considered statistically significant unless otherwise specified.

Potential confounders were selected based on a literature review and data availability. We applied a directed acyclic graph to illustrate the hypothesis of the relationship between exposure, outcomes, and covariates (Figure C.1). Sex and potential confounders including municipality at birth, household socio-economic status (or parents’ occupation), study subjects’ occupation at 24 years of age, any parent born outside of Sweden, body mass index (BMI), maternal smoking during pregnancy, parental smoking, individual smoking, time of the day for the urine collection, family history of asthma and birth season were examined but not included into the final model as they did not significantly (>10%) change the estimates, based on the metabolome model. In the analyses of the impact of air pollution during the first year of life and one year prior to the 4-year sampling, the final models were adjusted for parents’ education at baseline, while the model for air pollution during the prior year before the 24-year sampling included study subjects’ education level at 24 years of age as a covariate.

Sex and overweight status were each further examined as effect modifiers by additionally including an interaction term in the metabolite-specific model. For metabolites (excluding xenobiotics) that were significantly associated with at least one air pollutant in the main model (*p* < 0.05) and in the interaction model (p of the interaction term <0.05), we applied a stratified analysis.

### Sensitivity analysis

To explore to what extent the metabolite-specific associations may be influenced by lifestyle factors, we conducted sensitivity analyses additionally adjusting for smoking (maternal smoking during pregnancy and/or parental smoking for early life exposure, individual smoking for prior year exposure in 24-year samples), caffeine (biomarker of caffeine intake [37]) and ethyl-glucuronide (biomarker of alcohol consumption in 24-year samples [38]). Additionally, we conducted sensitivity analysis adjusted for occupation (parents’ occupation status at 3 months for early life exposure, individual occupation for prior year exposure in 24-year samples) instead of education. Pearson’s correlation test was used to compare the estimates in sensitivity analyses with the main model, and the Kolmogorov-Smirnov test was used to compare the distribution of *p* values in sensitivity analyses with the main model. Potential influence of a more allergic symptoms- asthma- and low lung function- enriched population was explored 1) in sensitivity analysis adjusting for asthma and allergic symptoms in 4-year follow-up and adjusting for asthma and diagnosis of low lung function in 24-year follow-up, as well as 2) in a weighted analysis [39] based on inverse probability of selection weighting (i.e., assigning weights based on prevalence ratios of allergic symptoms, allergy, and low lung function in the population-based BAMSE birth cohort vs. our study population).

### Metabolic pathway enrichment analysis

To explore biological functions and molecular mechanisms associated with significant metabolic features, a pathway enrichment analysis was carried out using Metaboanalyst 6.0 [40]. We selected metabolic features at the top 10% based on the rank of the absolute value of the percentage change in predicted median and *p*-value for pathway analysis. For the annotated metabolites, compound names and the Kyoto Encyclopedia of Genes and Genomes (KEGG) database were used [41]. For untargeted features, pathway analysis was conducted using Mummichog (v.2.0.1), a bioinformatics platform that infers and categorizes functional biological activity directly from mass spectrometry output, without prior metabolite validation [42, 43]. The retention time, *m/z* value, and the results of association analysis were input into the Mummichog. To further minimize the possibility of false positive discovery, candidate pathways were also inspected using the most common forms out of the 16 standard adduct forms in Mummichog [43]. For untargeted analyses, the pathway analyses were based on a Mummichog curated database originated from KEGG, Biochemical Genetic and Genomic knowledgebase of large-scale metabolic reconstructions (BiGG), Edinburgh Human Metabolic Network (Edinburgh Model), and a community-driven consensus reconstruction of human metabolism (Recon2) [43].

To assess the robustness of our untargeted approach, we performed sensitivity analyses using alternate parameters (5% significance cutoff; 2 adduct forms: [M + H]+ and [M–H]–). While these conditions yielded metabolite networks more closely aligned with our annotated results, it is not largely different from our current analyses, and therefore we retained the 10% cutoff and 16 adduct forms for untargeted analyses to maximize feature coverage in hypothesis generation, consistent with mummichog’s design for high-quality untargeted discovery [42].

### Genetic variants and air pollution interaction analyses on urine metabolites

Guided by the metabolomics findings, we conducted a post hoc analysis of the interaction between genetic variants and air pollution on the identified metabolites to facilitate the interpretation of potential mechanisms. The OpenTargets Genetics database was used to search for single-nucleotide polymorphisms (SNPs) previously associated with selected urine metabolites that showed significant associations with air pollution (*p* < 0.05) [44, 45]. Genotyping has been conducted in two subsets of BAMSE participants, referred to as “Waves” (further details described in Appendix A) [46]. The effect of the interaction of the selected genetic variants with prior-year and first-year-of-life air pollutants on the levels of each of those metabolites at 4 and 24 years of age was assessed through exponential regression models. Moreover, the winsorization of metabolite levels was carried out following the approach described above. For each metabolite, the regression included the allele dosages of each SNP, air pollution levels, and a term of interaction between the SNP and air pollution as independent variables. In this study, the term “sub-additive” refers to an interaction where the joint association is less than the sum of independent associations of air pollution exposure and the allele (effect or reference) showing a direction of association consistent with the effect of the air pollution. Two principal components of genetic ancestry, sex, parental education level at birth of the participant, and genotyping Wave were also added as covariates. The regressions evaluating the effect of the genetic interaction with prior-year air pollutants on metabolites measured at 24 years of age were adjusted by the participant’s education level at the time of the data collection visit instead. The evidence of significant interaction was considered after a FDR adjustment of 5% (FDR *p* < 0.05). The Benjamini-Hochberg method was applied across all genetic variants per metabolite and air pollutant.

Sensitivity analyses were carried out accounting for the effect of tobacco smoke exposure. In the case of first-year-of-life air pollutants, the association testing with metabolites at 4 and 24 years of age was further adjusted by maternal smoking during pregnancy and parental smoking during the first year of life. A variable related to individual active smoking habits was included in the evaluation of the effect of the interaction with exposure to prior-year air pollution on metabolites measured at 24 years.

### Ethics approval and consent to participate

This study was conducted in accordance with relevant guidelines and regulations. Ethical approvals for the BAMSE cohort and the analyses performed in this study were obtained from the Regional Ethics Review Board, Karolinska Institutet, Stockholm, Sweden DNR: 98-175, 2016/1380-31/2. All caregivers and adult study subjects provided written informed consent.

## Results

### Descriptive statistics of the study population

The study population consisted of 880 subjects: 614 subjects at the 4-year follow-up and 846 subjects at the 24-year follow-up. Two 4-year samples were excluded based on the PCA analyses as potential measurement errors, resulting in 612 4-year samples and 578 paired samples (samples from the same individual at 4 and 24 years). The distribution of air pollution exposures (PM_10_, PM_2.5,_ and NO_x_), and relevant covariates at 4-year and 24-year follow-ups are described in Table 1. The background characteristics were generally comparable between the two follow-ups. A comparison between the study population and the original cohort is given in Table B.1, which shows that the study population did not differ from the complete cohort regarding sex and socioeconomic factors. As expected given the study design, the present study population had higher prevalence of symptoms of allergic disease at the 4-year follow-up (53% *vs*. 36%), higher prevalence of asthma at the 4-year (13% *vs*. 7%) and 24-year follow-ups (31% *vs*. 11%), and higher prevalence of low lung function at the 24-year follow-up (19% vs. 7%) compared to the BAMSE cohort at respective time point (Table B.1). The air pollution levels during the last year prior to biosampling were higher at the 4-year follow-up than the 24-year follow-up, particularly for NO_x_ (median (q25, q75): 21.6 (13.9, 30.0) *vs*. 9.5 (6.5, 13.4)) (Table 1). Correlations were strong among air pollutants during the same time window (Spearman’s correlation mostly >0.9) (Table B.2). The distributions of 260 annotated metabolites are described in Table B.3. The annotated metabolites had a low percentage of missing (<9% for each metabolite at each follow-up) (Table B.3).

**Table 1:** Descriptive characteristics of involved subjects in the 4-year follow-up and 24-year follow-up from the Swedish BAMSE cohort.

Characteristics	4-year follow-up	24-year follow-up
	*N* = 612	*N* = 846
Age at the biosampling date, mean ± sd, years	4.3 ± 0.2	22.7 ± 0.6
Female sex	325 (53%)	470 (56%)
Body mass index, mean ± sd	16.2 ± 1.3^a^	23.5 ± 4.1
Any parent born outside of Sweden	111 (19%)	167 (21%)
**Air pollution:**		
PM_10_ 0–1 year, ^b^median (q25, q75), μg/m^3^	14.4 (13.2, 16.0)	14.3 (13.1, 15.9)
PM_10_ during the prior year, ^c^median (q25, q75), μg/m^3^	12.7 (11.4, 13.8)	11.6 (10.8, 12.1)
PM_2.5_ 0–1 year, ^b^median (q25, q75), μg/m^3^	8.8 (8.3, 9.4)	8.7 (8.3, 9.4)
PM_2.5_ during the prior year, ^c^median (q25, q75), μg/m^3^	7.3 (6.8, 7.8)	5.2 (4.9, 5.5)
NO_x_ 0–1 year, ^b^median (q25, q75), μg/m^3^	28.9 (18.5, 41.4)	28.6 (18.4, 40.7)
NO_x_ during the prior year, ^c^median (q25, q75), μg/m^3^	21.6 (13.9, 30.0)	9.5 (6.5, 13.4)
**Parental education at birth**^d^**:**		
Elementary school	14 (2%)	17 (2%)
High School	273 (45%)	357 (42%)
University	324 (53%)	470 (56%)
**Education at the current follow-up**^e^**:**		
Elementary school or high school	NA	525 (62%)
University, college, or folk high school	NA	318 (38%)
**Diagnosis of asthma by the current follow-up**^f^:		
No	526 (86%)	580 (69%)
Yes	82 (13%)	265 (31%)
**Smoking/snuff use in the household:**		
Maternal smoking during pregnancy	82 (13%)	109 (13%)^g^
Any of the parents smoking during the first year of life	145 (24%)	196 (23%)
Current smoking	NA	166 (20%)
Current snuff use	NA	116 (14%)

*NA* Not applicable.

^a^*N* = 609 for 4-year follow-up due to missing data.

^b^Time-weighted average exposure during 0-1 years of age; *N* = 610 for 4-year follow-up and 842 for 24-year follow-up due to missing data.

^c^Time-weighted average exposure during the last year before the biosampling date; *N* = 591 for 4-year follow-up and 561 for 24-year follow-up due to missing data.

^d^Highest education level of the household at baseline, based on maternal and paternal education level; *N* = 611 for 4-year follow-up and 844 for 24-year follow-up due to missing data.

^e^*N* = 843 for 24-year follow-up due to missing data.

^f^*N* = 608 for 4-year follow-up and 845 for 24-year follow-up due to missing data.

^g^*N* = 845 for 24-year follow-up due to missing data.

### Associations between air pollution and urine metabolome

At the level of all 260 annotated metabolites, higher air pollution exposure was overall associated with higher levels of urinary metabolites. The estimated median fold change at 4 years of age for 1 IQR (2.8 μg/m^3^) increase in PM_10_ during the first year of life was 1.017 (*p* = 2.06e−09), and during the prior year 1.017 (*p* = 1.42e−07), also consistent for PM_2.5_ and NO_x_ (Table 2). To interpret it, every 2.8 μg/m^3^ increment in first-year PM_10_ is associated with 1.7% increase in the abundance of urinary metabolites (an average trend for these 260 metabolites). The estimated variance of the random effect was 2.6 (95% Confidence Interval (CI): 2.3, 3.0), which suggests that the specific metabolites are heterogeneous (Intraclass Correlation Coefficient (ICC) = 56.6%). Similarly, at the level of all 260 annotated metabolites, both early life and prior year air pollution exposure were associated with higher levels in urinary metabolites at 24 years (Table 2). In each model based on the 24-year follow-up data, the estimated variance of the random effect also indicated heterogeneity (ICC = 51.2%). Parametric models using other common distributions showed similar model fit (Table B.4). Note that on an individual metabolite basis, some metabolites may increase, others may decrease or show no significant association with air pollutants, but overall exposure to higher air pollution levels were associated with higher levels of urinary metabolites at both 4 and 24 years, driven by an imbalance in upregulated versus downregulated associations, suggesting systematic metabolic perturbation.

**Table 2:** Associations between the abundance of the urine metabolome (260 metabolites) and air pollution exposure during different time periods.

Model^a^	Estimated median fold change^b^	*P* value
4-year follow-up (random effect of 6 models are 2.6 (95% CI: 2.3, 3.0), ICC = 56.6%)		
PM_10_ 0-1 year	1.017	2.06e−09
PM_10_ during the prior year	1.017	1.42e−07
PM_25_ 0-1 year	1.017	1.18e−08
PM_25_ during the prior year	1.012	9.75e−05
NO_x_ 0-1 year	1.018	1.88e−08
NO_x_ during the prior year	1.021	1.23e−06
24-year follow-up (random effect of 6 models are 2.1 (95% CI: 1.8, 2.4), ICC = 51.2%)		
PM_10_ 0-1 year	1.012	6.36e−07
PM_10_ during the prior year	1.027	1.43e−08
PM_25_ 0-1 year	1.009	2.48e−04
PM_25_ during the prior year	1.015	1.70e−03
NO_x_ 0-1 year	1.015	1.85e−07
NO_x_ during the prior year	1.046	1.41e−10

^a^The results are adjusted for parents’ education for all models at the 4-year follow-up and for models with air pollutants at the 1^st^ year of life at the 24-year follow-up, and adjusted for individual education for models with air pollutants at the prior year of biosampling at the 24-year follow-up.

^b^The estimates are presented per 1 IQR increase in air pollution exposure level, corresponding to 2.8, 1.1, and 22.9 μg/m^3^ for PM_10_, PM_2.5,_ and NO_x_, respectively.

### Associations between air pollution and specific metabolites at 4 years of age

**Fig. 2:**
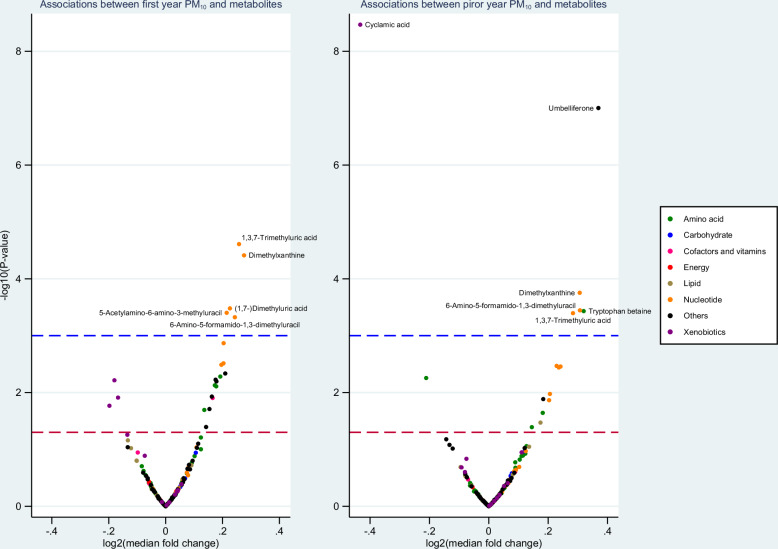
Associations between PM_10_ and 260 annotated metabolites at 4 years of age. The blue line indicates FDR *p* = 0.05. The red line indicates *p* = 0.05. Dimethylxanthine indicates 1,7-dimethylxanthine. The results are extracted from exponential regression models, adjusted for parents’ education at 3 months. For each metabolite, observations above the 99^th^ percentile value were replaced with the 99th percentile value.

At 4 years of age, air pollution exposure both during the first year of life and the year prior to biosampling was associated with an increase in eight nucleotide-related compounds (fold change: 1.12 to 1.36; seven of eight FDR significant with at least one of the three exposures: PM_10_, PM_2.5_ or NO_x_; Fig. 2, Fig. D.1, D.2). These include six purines (1,7-dimethylxanthine, 1,3,7-trimethyluric acid, 1-methylxanthine, (1,7-)dimethyluric acid, 1,3-dimethyluric acid, and methyluric acid) and two pyrimidine derivatives (6-amino-5-formamido-1,3-dimethyluracil and 5-acetylamino-6-amino-3-methyluracil) (Table 3, Tables B.5, B.6). For example, compared with children exposed to median levels of PM_10_, PM_2.5_, or NO_x_ during their first year of life, one IQR increase in air pollution exposure was associated with a 1.20 to 1.22-fold increase in estimated median of 1,7-dimethylxanthine (FDR p < 0.05).

**Table 3:** Associations between air pollution and annotated metabolites at age 4 years.

Metabolite	Super pathway	Associated exposures	First year exposure:	Prior year exposure:
			range of median fold change	range of median fold change
1,7-Dimethylxanthine	Nucleotide derivatives	First year PM_10_*, PM_2.5_*, NO_x_*; Prior year PM_10_*, PM_2.5_*, NO_x_*	1.20, 1.22	1.22, 1.32
6-Amino-5-formamido-1,3-dimethyluracil	Nucleotide derivatives	First year PM_10_*, PM_2.5_*, NO_x_*; Prior year PM_10_*, PM_2.5_*, NO_x_*	1.17, 1.21	1.22, 1.34
1,3,7-Trimethyluric acid	Nucleotide derivatives	First year PM_10_*, PM_2.5_*, NO_x_*; Prior year PM_10_*, PM_2.5_*, NO_x_*	1.16, 1.25	1.20, 1.36
1-Methylxanthine	Nucleotide derivatives	First year PM_10_, PM_2.5_, NO_x_; Prior year PM_10_, PM_2.5_, NO_x_*	1.15, 1.16	1.16, 1.27
(1,7-)Dimethyluric acid	Nucleotide derivatives	First year PM_10_*, PM_2.5_*, NO_x_*; Prior year PM_10_, PM_2.5_, NO_x_*	1.15, 1.19	1.15, 1.28
5-Acetylamino-6-amino-3-methyluracil	Nucleotide derivatives	First year PM_10_*, PM_2.5_, NO_x_*; Prior year PM_10_, PM_2.5_, NO_x_*	1.12, 1.20	1.14, 1.31
1,3-Dimethyluric acid	Nucleotide derivatives	First year PM_10_, PM_2.5_, NO_x_*; Prior year PM_10_, PM_2.5_, NO_x_*	1.13, 1.18	1.13, 1.27
Methyluric acid	Nucleotide derivatives	First year PM_10_, PM_2.5_, NO_x_; Prior year PM_10_, PM_2.5_, NO_x_	1.15, 1.15	1.12, 1.24
2-O-Methylinosine	Nucleotide derivatives	Prior year NO_x_	1.06, 1.07	1.05, 1.16
Anserine	Amino acid derivatives	First year PM_10_, PM_2.5_, NO_x_; Prior year PM_10_, PM_2.5_, NO_x_	1.11, 1.15	1.13, 1.19
Succinylacetone	Amino acid derivatives	First year PM_10_, PM_2.5_, NO_x_; Prior year PM_10_	1.13, 1.16	1.09, 1.11
Furosine	Amino acid derivatives	First year PM_10_, PM_2.5_, NO_x_	1.09, 1.10	1.05, 1.07
N-Methyl-proline	Amino acid derivatives	First year PM_10_, PM_2.5_, NO_x_	1.12, 1.15	1.04, 1.10
Tryptophan betaine	Amino acid derivatives	Prior year PM_10_*, PM_2.5_, NO_x_*	1.09, 1.13	1.21, 1.47
cis-Urocanic acid	Amino acid derivatives	Prior year PM_10_, PM_2.5_, NO_x_	0.97, 1.05	0.83, 0.86
Methyl-histidine	Amino acid derivatives	Prior year PM_2.5_	1.08, 1.11	1.07, 1.14
Azelaic acid	Lipid derivatives	Prior year PM_10_, PM_2.5_, NO_x_	1.06, 1.08	1.13, 1.19
Suberic acid	Lipid derivatives	Prior year NO_x_	1.04, 1.04	1.08, 1.16
Stearic acid	Lipid derivatives	First year NO_x_; Prior year NO_x_	0.89, 0.94	0.86, 0.97
Panthenol	Cofactors and vitamins	First year PM_10_, PM_2.5_, NO_x_	1.12, 1.14	1.05, 1.12
Umbelliferone	Others	First year PM_10_, PM_2.5_, NO_x_*; Prior year PM_10_*, PM_2.5_*, NO_x_*	1.10, 1.23	1.29, 1.35
4-Hydroxycoumarin	Others	First year PM_10_, PM_2.5_, NO_x_; Prior year PM_10_, PM_2.5_, NO_x_	1.12, 1.14	1.14, 1.16
Quinic acid	Others	First year PM_10_; Prior year PM_10_, PM_2.5_	1.10, 1.12	0.87, 0.91
Ferulic acid 4-sulfate	Others	First year PM_10_, PM_2.5_, NO_x_	1.13, 1.15	1.04, 1.06
2-Benzoxazolol	Others	First year PM_10_, PM_2.5_, NO_x_	1.13, 1.16	0.98, 1.01
Adipic Acid	Others	First year PM_10_, PM_2.5_	1.10, 1.13	1.00, 1.06
Trimethylamine N-Oxide	Others	Prior year PM_2.5_	0.94, 0.95	0.90, 0.92
2-Piperidone	Others	Prior year NO_x_	0.93, 0.96	0.86, 0.92
Cyclamic acid	Xenobiotics	First year PM_10_, PM_2.5_, NO_x_; Prior year PM_10_*, PM_2.5_*, NO_x_*	0.86, 0.91	0.68, 0.74
Saccharin	Xenobiotics	First year PM_10_, PM_2.5_, NO_x_	0.86, 0.89	0.89, 0.95
Acesulfame	Xenobiotics	First year PM_2.5_; Prior year PM_2.5_	0.91, 0.95	0.92, 0.95
Acetaminophen	Xenobiotics	First year PM_10_, NO_x_	0.83, 0.91	1.08, 1.09

Footnote: The results are extracted from exponential regression models, adjusted for parents’ education at 3 months. For each metabolite, observations above the 99th percentile value were replaced with the 99th percentile value before modeling. Associated exposures are presented when nominal *p*-value was <0.05. *It indicates FDR *p*-value < 0.05. The range of median fold change is presented irrespective of significance.

Further, first-year or prior-year air pollution exposure was mostly nominally associated with seven amino acid derivatives (fold change 0.83 to 1.47, *e.g*., anserine, succinylacetone, furosine, N-methyl-proline, tryptophan betaine, cis-urocanic acid, and methyl-histidine), three fatty acids (fold change 0.86 to 1.19, e.g., azelaic acid, suberic acid, and stearic acid), one vitamin panthenol (fold change: 1.05 to 1.14), and eight other metabolites (fold change: 0.86 to 1.35, *e.g*., umbelliferone, 4-hydroxycoumarin, quinic acid, ferulic acid 4-sulfate, 2-benzoxazolol, adipic acid, trimethylamine N-Oxide, 2-piperidone), as well as four xenobiotics (fold change: 0.68 to 1.09, *e.g*., cyclamic acid, saccharin, acesulfame, and acetaminophen) (Table 3).

Some xenobiotics showed a higher effect size (fold change: 0.68 to 1.09) than most other types of metabolites. However, when conducting a bivariate visual assessment of the associations between metabolites and air pollution exposures, xenobiotics were found to have a distribution with most values closer to zero and less clear distinction across quartiles of air pollution compared with other types of metabolites (Fig. E.1-E.32), implying that xenobiotics were more prone to the effects of extreme values.

**Fig. 3:**
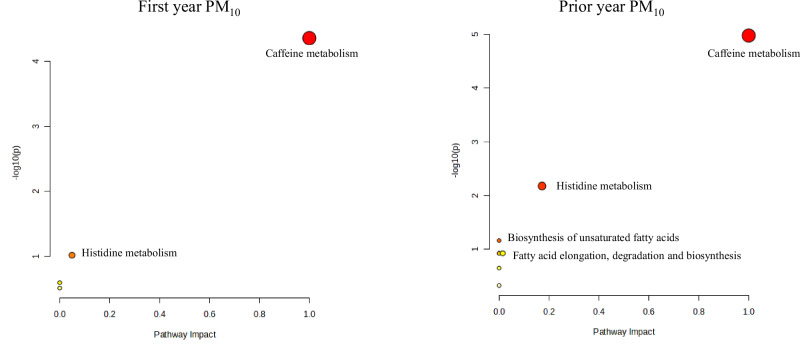
Topology plots of top-enriched pathways at age 4, associated with PM_10._ The Y-axis represents the negative logarithm of the *p*-value (base 10 logarithm) derived from the enrichment analysis. The X-axis indicates the structural impact of PM_10_-associated metabolites within the enriched pathways, calculated based on the cumulative importance of all significant metabolites in the pathway. The bubble size reflects the impact value, while the bubble color indicates the significance of the enrichment.

In the enrichment analyses, the top 10% enriched metabolic pathways based on fold change were very similar to those based on *p*-value. Figure 3 shows the top pathways associated with air pollution at age 4, using PM_10_ as an example. Both first-year and prior-year exposures to PM_10_ were significantly associated with eight caffeine pathway metabolites (1,7-dimethylxanthine, 6-amino-5-formamido-1,3-dimethyluracil, 1,3,7-trimethyluric acid, 1-methylxanthine, 1,7-dimethyluric acid, 5-acetylamino-6-amino-3-methyluracil, 1,3-dimethyluric acid, and methyluric acid). Evidence was not strong enough to support the association between air pollution and caffeine itself (fold change: 1.07-1.15, p values ranging from 0.06 to 0.13, with five out of six p values between 0.09 and 0.13, for any first-year or prior year air pollution exposure). Prior-year exposure to PM_10_ was further associated with two histidine pathway metabolites (anserine and methyl-histidine), as well as weakly associated with some fatty acid-related pathways. Caffeine metabolism and fatty acid-related pathways were also found by supplementary enrichment pathway analyses based on the untargeted metabolomics feature datasets (Table B.7). A mechanistic plot of the hypothesized relationship between air pollution and caffeine metabolism can be found in Fig. C.2.

In the sensitivity analysis, the eight metabolites related to caffeine metabolism remained similar in magnitude and significance when adjusting for caffeine (Table B.8). Additional adjustment for maternal smoking during pregnancy and parental smoking during the first year of life, or adjustment for occupation instead of education, did not significantly change the associations (Tables B.9, B.10). Sensitivity analyses adjusting for asthma and allergic symptoms did not change the estimates, although in a weighted analysis the effect estimates were somewhat decreased (Fig. C.3).

### Associations between air pollution and specific metabolites at 24 years of age

**Fig. 4:**
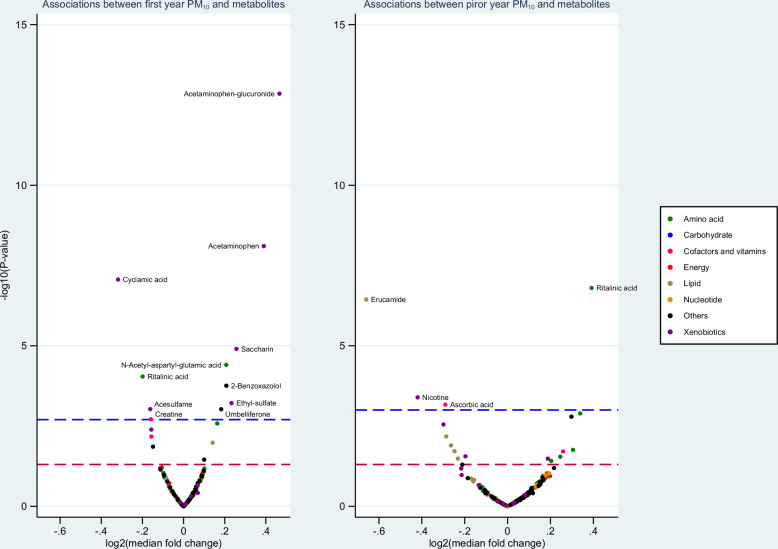
Associations between PM_10_ and 260 annotated metabolites at 24 years of age. The blue line indicates FDR *p* = 0.05, and the red line indicates nominal *p* = 0.05. All results are adjusted for parents’ education at 3 months (for first-year exposure) or individual education (for prior-year exposure). For each metabolite, observations above the 99th percentile value were replaced with the 99th percentile value.

At age 24, prior year air pollution exposure was associated with a decrease in levels of six lipid derivatives (three were FDR significant with at least one of the exposures PM_10_, PM_2.5_ or NO_x_, Fig. 4, Figures D.3, D.4), particularly long-chain fatty acids (Table 4, Tables B.11, B.12). Compared with young adults exposed to median level of PM_10_, PM_2.5_ or NO_x_, during the prior year of life, one IQR increase in air pollution exposure was associated with 0.82 to 0.76-fold decrease in estimated median of stearic acid (for associations with NO_x_ and PM_10_, nominal *p* values < 0.05; for PM_2.5_, FDR *p* < 0.05). Furthermore, one IQR increase in prior year air pollution exposure was associated with a decrease in palmitic acid (estimated median fold change: 0.76 to 0.83), oleic acid (fold change: 0.72 to 0.84), linoleic acid (fold change: 0.76 to 0.85), hexadecenoic acid (0.84 to 0.89), and erucamide (fold change: 0.62 to 0.77), a derivative of the long-chain fatty acid erucic acid.

**Table 4:** Associations between air pollution and annotated metabolites at age 24 years.

Metabolite	Super pathway	Associated exposures	First year exposure:	Prior year exposure:
			range of median fold change	range of median fold change
Carnosine	Amino acid derivatives	Prior year PM_10_, PM_2.5_, NO_x_*	1.02, 1.04	1.26, 1.38
N-Acetyl-aspartyl-glutamic acid	Amino acid derivatives	First year PM_10_*, PM_2.5_*, NO_x_*; Prior year PM_10_, PM_2.5_*, NO_x_	1.15, 1.17	1.13, 1.31
N-Acetyl-tyrosine	Amino acid derivatives	First year PM_2.5_; Prior year PM_2.5_*	0.91, 0.97	1.15, 1.32
Alliin	Amino acid derivatives	Prior year PM_10_, PM_2.5_	1.03, 1.07	1.23, 1.29
3-Hydroxyanthranilic acid	Amino acid derivatives	Prior year PM_10_, PM_2.5_, NO_x_	0.98, 1.00	1.19, 1.27
Anserine	Amino acid derivatives	Prior year PM_2.5_, NO_x_	1.00, 1.02	1.11, 1.43
N-Cinnamoylglycine	Amino acid derivatives	Prior year PM_2.5_	1.02, 1.03	1.12, 1.17
Betonicine	Amino acid derivatives	Prior year PM_10_, NO_x_	0.99, 1.00	1.12, 1.26
Taurine	Amino acid derivatives	First year NO_x_	0.92, 0.93	0.89, 0.94
Ritalinic acid	Amino acid derivatives	First year PM_10_*, PM_2.5_*, NO_x_*; Prior year PM_10_*, NO_x_*	0.84, 0.88	1.08, 1.77
Theanine	Amino acid derivatives	First year PM_10_, PM_2.5_, NO_x_*	1.12, 1.15	0.98, 1.04
Hexitol (Sorbitol, Mannitol, Galactitol)	Carbohydrate	Prior year PM_2.5_	0.99, 1.02	1.11, 1.20
Ascorbic acid	Cofactors and vitamins	Prior year PM_10_*, PM_2.5_*, NO_x_	0.92, 0.93	0.76, 0.82
Nicotinic acid	Cofactors and vitamins	Prior year PM_2.5_	0.98, 1.02	1.14, 1.25
Pyridoxine	Cofactors and vitamins	First year PM_10_, PM_2.5_*, NO_x_; Prior year PM_10_, PM_2.5_, NO_x_	0.88, 0.90	1.20, 1.40
Creatine	Cofactors and vitamins	First year PM_10_*, PM_2.5_*, NO_x_*	0.87, 0.90	0.84, 0.98
Stearic acid	Lipid derivatives	Prior year PM_10_, PM_2.5_*, NO_x_	0.94, 0.98	0.76, 0.82
Palmitic acid	Lipid derivatives	Prior year PM_10_, PM_2.5_*, NO_x_	0.95, 0.98	0.76, 0.83
Oleic acid	Lipid derivatives	Prior year PM_10_, PM_2.5_, NO_x_	0.95, 0.99	0.72, 0.84
Linoleic acid	Lipid derivatives	Prior year PM_10_, PM_2.5_, NO_x_	0.95, 0.99	0.76, 0.85
Erucamide	Lipid derivatives	First year PM_2.5_; Prior year PM_10_*, PM_2.5_, NO_x_*	0.92, 0.98	0.62, 0.77
Hexadecenoic acid	Lipid derivatives	Prior year PM_2.5_	1.03, 1.05	0.84, 0.89
Azelaic acid	Lipid derivatives	First year PM_10_, PM_2.5_, NO_x_	1.10, 1.12	0.96, 0.98
Tartaric acid	Others	Prior year PM_10_, PM_2.5_, NO_x_	0.96, 0.99	1.23, 1.36
Umbelliferone	Others	First year PM_10_*, PM_2.5_*, NO_x_*; Prior year PM_2.5_	1.13, 1.16	0.83, 1.25
3-Hydroxypyridine	Others	Prior year PM_2.5_	0.93, 0.94	1.16, 1.26
2-Hydroxypyridine	Others	Prior year PM_2.5_	0.99, 1.01	1.11, 1.20
Homogentisic acid	Others	First year PM_10_, PM_2.5_, NO_x_; Prior year NO_x_	0.88, 0.91	0.74, 1.05
Lactic acid	Others	First year PM_10_	1.04, 1.07	0.79, 0.97
2-Benzoxazolol	Others	First year PM_10_*, PM_2.5_*, NO_x_*	1.16, 1.17	0.99, 1.04
Saccharin	Xenobiotics	First year PM_10_*, PM_2.5_*, NO_x_*; Prior year PM_10_, PM_2.5_	1.19, 1.23	0.79, 0.86
Acetaminophen-glucuronide	Xenobiotics	First year PM_10_*, PM_2.5_*, NO_x_*; Prior year PM_10_, NO_x_	1.24, 1.44	0.76, 1.25
Ethyl-glucuronide	Xenobiotics	First year NO_x_; Prior year PM_10_, PM_2.5_	1.05, 1.13	0.85, 1.12
Nicotine	Xenobiotics	First year PM_10_, PM_2.5_*, NO_x_; Prior year PM_10_*, PM_2.5_, NO_x_	0.89, 0.90	0.75, 0.80
Acetaminophen	Xenobiotics	First year PM_10_*, PM_2.5_*, NO_x_*; Prior year PM_2.5_	1.18, 1.38	0.80, 0.91
Acesulfame	Xenobiotics	First year PM_10_*, PM_2.5_*, NO_x_*	0.86, 0.89	0.91, 0.93
Ethyl-sulfate	Xenobiotics	First year PM_10_*, PM_2.5_*, NO_x_*; Prior year PM_10_, NO_x_*	1.18, 1.25	1.07, 1.53
Cyclamic acid	Xenobiotics	First year PM_10_*, PM_2.5_*, NO_x_*	0.73, 0.80	1.00, 1.05

Footnote: The results are extracted from exponential regression models, adjusted for parents’ education at 3 months (for first year exposure) or individual education (for prior year exposure). For each metabolite, observations above the 99^th^ percentile value were replaced with the 99th percentile value before modeling. Associated exposures are presented when nominal *p*-value was <0.05. *It indicates FDR *p*-value < 0.05. The range of median fold change is presented irrespective of significance.

Prior year air pollution was also associated with an increase in nine amino acid derivatives. In addition, first-year or prior-year air pollution exposure was associated with eight xenobiotics, one carbohydrate, four cofactors and vitamins, and seven other metabolites (Table 4). The associations with the eight caffeine-pathway metabolites found in 4-year samples were not observed in the analyses with 24-year samples. Similar to the results at age 4, when conducting a bivariate visual assessment of the associations between metabolites and air pollution exposures, the distribution of xenobiotics generally indicated effects of a few extreme values (Figures F.1–F.38).

**Fig. 5:**
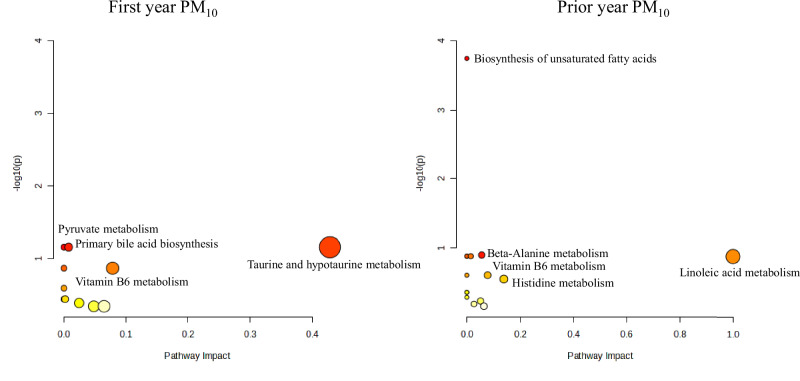
Topology plots of top enriched pathways at age 24, associated with PM_10_. The Y-axis represents the negative logarithm of the *p*-value (base 10 logarithm) derived from the enrichment analysis. The X-axis indicates the structural impact of PM_10_-associated metabolites within the enriched pathways, calculated based on the cumulative importance of all significant metabolites in the pathway. The bubble size reflects the impact value, while the bubble color indicates the significance of the enrichment.

In the enrichment analyses for 24-year samples, the strongest enriched pathways were also very similar based on fold change and *p*-value. Figure 5 shows the top most significantly enriched metabolic pathways associated with air pollution exposure at age 24, using PM_10_ as an example. Driven by four fatty acids (stearic acid, palmitic acid, oleic acid, and linoleic acid), prior-year exposure to PM_10_ at age 24 was associated with the biosynthesis of unsaturated fatty acids. Fatty acid-related pathways were also supported by enrichment pathway analyses based on the untargeted features (Table B.13).

In the sensitivity analyses, the direction and magnitude of the associations were not significantly changed after additionally adjusting for smoking, caffeine, and ethyl-glucuronide, or adjusting for occupation, respectively (Tables B.14–B.17). In both the 4-year and the 24-year samples, pathway enrichment analyses based on associations between metabolites and PM_2.5_ or NO_x_ showed similar findings compared with PM_10_ (Figures G.1–G.4). Sensitivity analyses adjusting for asthma and low lung function or using a weighted approach did not alter the results (Figure C.3).

Results of stratified analysis by BMI or sex are presented in Tables B.18–B.25. Both BMI and sex showed some effect modification. In particular, in the 24-year samples, individuals with overweight showed an indication of upregulated long-chain fatty acids, albeit not statistically significant, while normal-weight or underweight individuals showed downregulated long-chain fatty acids (mostly FDR significant) in association with prior year air pollution (Table B.21).

### Genetic variant - air pollution interaction on caffeine or coumarin pathway metabolites

To better understand if the identified caffeine pathway metabolites were more related to alterations in pathways instead of behavioral factors, we conducted post hoc analysis by including metabolism-related genetic variants. The interaction of genetic variants and air pollutants on the levels of metabolites potentially derived from caffeine or coumarin was explored. Metabolites from both caffeine and coumarin were included since they could be derived through the action of the same cytochrome P450 enzyme (CYP1A2) and have shared metabolic pathways [47–50]. Ten metabolites nominally associated with at least one air pollutant were included (1,7-dimethylxanthine, 1,3,7-trimethyluric acid, 1-methylxanthine, (1,7-)dimethyluric acid, 1,3-dimethyluric acid, methyluric acid, 6-amino-5-formamido-1,3-dimethyluracil, 5-acetylamino-6-amino-3-methyluracil, umbelliferone, and 4-hydroxycoumarin).

Ten SNPs previously associated with caffeine-derived metabolites were available in BAMSE and selected for the analyses (Table B.26). Among these, seven SNPs were found to be associated (*p* < 0.05) with the 4-year levels of at least one of the ten metabolites assessed (Table B.27). Four of these SNPs also showed significant associations at 24 years of age (Table B.28, Figs. H.1–H.10).

**Fig. 6:**
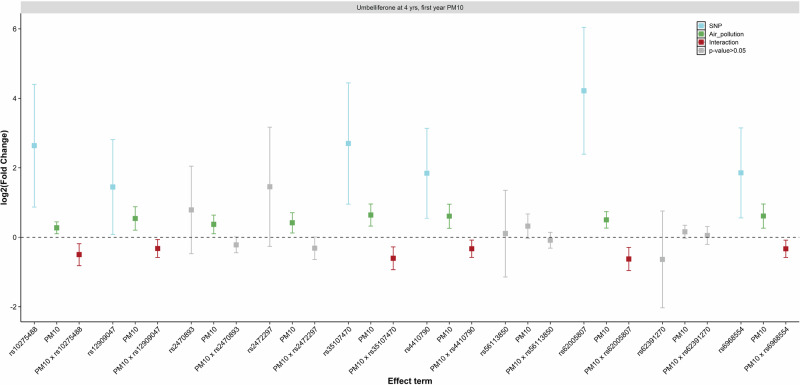
Interaction between PM_10_ exposure during the first year of life and caffeine metabolism-related SNPs, in relation to umbelliferone at 4 years of age. Association analyses were performed in 473 children with available metabolite levels, air pollution exposure, and genome-wide genotype data. Log2 scale of the estimated fold change and the estimated 95% CI are shown on the *y*-axis for the SNP, air pollutant, and interaction terms (*x*-axis).

Further analyses showed significant interactions between caffeine metabolism-associated genetic variants and air pollutants on caffeine and coumarin-derived metabolites in urine at 4 years of age. Taking PM_10_ as an example, six of the SNPs showed significant interactions with exposure during the first year of life on several of the evaluated metabolites at age 4 years (Table B.29). The highest number of significant interactions was observed on umbelliferone levels. Sub-additive interaction effects were consistently observed between caffeine metabolism-associated SNPs and both first-year-of-life and prior-year PM_10_ exposure on umbelliferone levels (Fig. 6, Figure H.11, Table B.29, and Table B.30). A similar pattern was observed in metabolites measured in urine samples collected at the 24-year follow-up (Tables B.31–B.32). When compared to the results with PM_10_, similar results were observed for the interaction of genetic variants with exposure to PM_2.5_ or NO_x_ on urine metabolites (Tables B.33–B.40).

Comparing the distribution of *p*-values of the interaction between PM_10_ and SNPs on caffeine and coumarin-derived metabolites at 4 and 24 years of age suggests that the results were more robust in children (Figure H.12). In the sensitivity analysis, the identified interactions remained similar in effect magnitude and significance when adjusting for smoking (Tables B.41–B.44).

## Discussion

We assessed the associations between air pollution exposure (during the first year of life as well as during the year prior to biosampling) and the metabolite levels measured in urine samples at 4 and 24 years of age. We found that preceding PM_10_, PM_2.5_, and NO_x_ exposures were strongly associated with higher levels of the 260 annotated urinary metabolites, although there was a heterogeneity of metabolite-specific associations in terms of both direction and magnitude. The top enriched pathways primarily included caffeine metabolism and histidine metabolism at 4 years, and fatty acid metabolism at 24 years. Further, we observed interactions between air pollution exposures and caffeine-metabolism-related genetic variants on identified caffeine and coumarin metabolites, especially at the 4-year follow-up.

Despite comparatively low air pollution levels, a multitude of associations between air pollution exposure and adverse health effects have been observed in Stockholm. Air pollution exposure during infancy has been linked to lung function decrement in 6-month-old infants [51], as well as an elevated risk of allergy, asthma, and lung function impairment in children up to school age [52–55], adolescence [56, 57], and even early adulthood [58, 59]. It is therefore reasonable to hypothesize that molecular alterations occur from childhood to young adulthood in response to air pollution exposure.

At 4 years, we found that air pollution was associated with upregulated metabolites in the caffeine pathway, including higher levels of six purine derivatives and two pyrimidine derivatives. However, no significant association with caffeine itself was observed. Among previous studies in children, the most relevant study on medium-term prenatal exposure to air pollution also reported upregulated levels of hypoxanthine in serum samples of newborns [15]. This is a purine-derivative that is related to caffeine metabolism via the purine metabolism pathway and inhibition of xanthine oxidase [60]. In our study, the 4-year samples were collected during 1998-2001 (4-year follow-up). The samples had much lower levels of caffeine (fold change in median caffeine from 4-year samples to 24-year samples: 31.7) and 1,7-dimethylxanthine (fold change in median 1,7-dimethylxanthine from 4-year samples to 24-year samples: 9.5), compared with the 24-year samples. Therefore, if the 4-year-olds consumed caffeine, it would only be in small amounts. A United States population-based study on young children aged 2-5 years conducted during 1999–2002 estimated that 62.7% of the total population at this age consumed caffeine, though the intake was low (17.4 mg/day during 1999–2000 and 20.6 mg/day during 2001), compared with adults (142.9 mg/day during 1999–2000) [61]. In more recent population-based studies, the caffeine intake was 13.07 mg/day in a Korean population aged 3–5 years, whereas 36% of Canadian children aged 1-5 years drank caffeinated beverages and consumed 7 mg/day caffeine on average [62, 63]. The most common sources of caffeine for this age group are soda, tea, flavored dairy, and cocoa products [61, 62].

One potential explanation behind the association of caffeine metabolism with air pollution could have been that parents who live in areas with higher air pollution were more likely to give products that contain caffeine-related metabolites (e.g., chocolate, caffeine-containing soft drinks) due to the disproportionate distribution of socioeconomic factors [64, 65]. Nonetheless, after adjusting for socio-economic status-related variables (i.e., education and occupation), caffeine metabolism-related metabolites at age 4 were consistently associated with air pollution. Furthermore, metabolites of caffeine were associated with preceding air pollution exposure, but caffeine itself was not associated with air pollution. This leads us to believe that the observed associations are *not* related to caffeine intake, but rather to a difference in metabolism between children with varying levels of air pollution exposure.

Another plausible explanation of the association of caffeine metabolism with air pollution would be that air pollution affects how the human body metabolizes caffeine, particularly in young children who are more vulnerable to exposure. Air pollution was associated with increased metabolites from not only the caffeine pathway but also the coumarin pathway, potentially linked to CYP1A2 enzyme activity [47–50]. Significant interactions between air pollution and caffeine-metabolism-associated SNPs on caffeine and coumarin-derived metabolites were also observed, especially at the 4-year follow-up. For example, significant interaction with SNPs located at genes encoding the Aryl Hydrocarbon Receptor (AHR), which has been shown to regulate CYP1A2 expression and to be activated by exposure to environmental pollutants [66–69].

While direct evidence of air pollution’s effect on CYP1A2 activity is limited, research on related environmental pollutants provides some insights. Nicotine or tobacco smoke is known to accelerate the metabolism of caffeine in the human body, potentially via increased CYP1A2 enzyme activity [70]. In addition, urinary caffeine metabolites (*i.e*., 1,7-dimethylxanthine, theobromine, theophylline, 1-methyluric acid, and 5-acetylamino-6-amino-3-methyluracil) have previously been used as a proxy for CYP1A2 activity [71, 72]. For example, one study reported that smokers had 1.55-fold higher CYP1A2 activity (measured as the ratio of 1,7-dimethylxanthine/caffeine) compared to smoking abstainers [73]. Moreover, polycyclic aromatic hydrocarbons (PAHs), primarily generated by incomplete combustion of coal or biomass and tobacco smoking [74], not only can induce CYP1A2 activity [75], but also have been found to interact with genetic variants at genes encoding enzymes involved in the metabolic activation or detoxification of PAHs (e.g., CYP1A1, CYP1B1, GSTM1, GSTT2) [76, 77]. Therefore, carrying the effect allele of these variants might modify the effect of air pollutants and the subsequent disease risk. Our findings not only illuminate caffeine metabolism but also suggest that there are SNPs that may indicate genetic susceptibility to air pollution effects more broadly, which merits further investigation in future health outcomes studies.

The gene-environment interactions we demonstrate in this study suggest that the metabolic changes might be due to alterations in pathways rather than caused by behavioral or socio-economic factors. The sub-additive nature of this interaction compared to the individual effects of the air pollutant and genetic variant suggests that both air pollution and SNPs influence the same pathway, potentially including CYP1A2 activity, with their combined effect less than the sum of individual effects, consistent with enzyme saturation. It has also been shown that exposure to air pollution can influence DNA methylation and gene expression, with genetic variants influencing enzyme activity [4, 78].

The same association with caffeine metabolism was not found in the 24-year samples, which is consistent with previous epidemiological studies in adults. In a review of 32 studies on air pollution and metabolomics, only three studies reported significant associations with caffeine metabolism [14]. The association with caffeine metabolism in the 4-year samples rather than 24-year samples can be explained by potential biological differences. It is important to note that human metabolism undergoes substantial changes during growth and development. Further, young adults are likely exposed to more diverse lifestyle risk factors than children [79]. Therefore, the association between long-term air pollution and caffeine pathway metabolites at 24 years could be small in magnitude and easier blurred by unmeasured factors.

At 24 years, we found that prior-year air pollution was associated with downregulated fatty acid metabolism. Among the few long-term air pollution studies that have been conducted, one focused on young adults, while others focused on later adulthood and elderhood. They have consistently reported associations between long-term exposure to PM or NO_2_ and long-chain fatty acids, or with compounds containing long-chain fatty acid component, or with fatty-acid-related pathways [16–18, 20]. However, these previous studies observed inconsistent results on the direction of the associations between long-term air pollution exposure and long-chain fatty acids. A study in southern California suggested that prior-year air pollution exposure is related to an upregulated trend in fatty acids in young adults with a history of overweight or obesity [20]. However, among three other studies on older populations, two reported downregulation in lipids containing long-chain fatty acids [16, 17] and one reported both up-and down-regulation in some long-chain fatty acids [18]. This inconsistency may be due to the variation in BMI across the study populations. Consistent with the study on a population with high BMI (mean BMI = 29.6) [20], in our stratified analysis, an overweight subgroup showed a trend of upregulation (which did not reach significance, likely due to a combination of small effect size and low statistical power). In the normal or underweight stratum we demonstrated downregulation of these fatty acids.

An implication of this finding is that downregulated long-chain fatty acids may explain some adverse health outcomes of air pollution exposure. In their untargeted metabolomics study using a meet-in-the-middle approach, Jeong et al. found linoleic acid metabolism to be a mediator of the association between air pollution and both asthma and cardio-cerebrovascular disease [18]. Furthermore, previous studies on mice indicate that diesel exhaust reduces the levels of linoleic acid and oleic acid, affecting inflammation resolution and mitochondrial β-oxidation [80, 81].

For the downregulated long-chain fatty acids with prior-year air pollution in the 24-year samples, many of the associations were of small magnitude and nominal significance in the main analysis, which is expected. First, since urine is not the main route of fatty acid elimination, the use of non-targeted measurement is not optimal for hydrophobic compound separation and detection. Importantly, the small magnitude of detected associations is expected, because the BAMSE birth cohort is a generally healthy population living with lower air pollution exposure compared to most previous studies. Therefore, it is interesting that we still replicated athe ssociation between air pollution and fatty acids metabolism in urine in this cohort.

In our 24-year samples, we also found some weakly enriched pathways such as β-alanine metabolism (underlying metabolite: carnosine), as well as taurine and hypotaurine metabolism (underlying metabolite: taurine), that has been suggested by a previous long-term air pollution study [17].

According to recent reviews [5, 13, 14], air pollution has been consistently associated with perturbations in metabolites such as hypoxanthine, histidine, serine, aspartate, glutamate, taurine and creatine, and metabolic pathways involving glycerophospholipid-, pyrimidine-, methionine/cysteine-, tyrosine-, and tryptophan metabolism. However, most of the previous studies have analyzed metabolites in plasma and serum, exploring associations with short-term air pollution in smaller samples (mostly <200). When we compared the significant metabolites identified in our study, we observed some results consistent with previous findings, for example, downregulated levels of taurine and creatine. We also replicated association with histidine metabolism in the 4-year samples, which has been consistently associated with short-term air pollution. Other previously suggested metabolites—such as anserine, tyrosine, and uracil—were not associated with air pollution in our study, but we did observe association with their derivatives or chemically related compounds.

In the 24-year follow-up, the FDR significant metabolite-specific associations were largely represented by exogenous compounds, typically dietary or medication-related compounds or exogenous compounds that could be associated with lower socio-economy such as ethyl-glucuronide and nicotine, many of which were affected by a small group of individuals with high levels.

Our study fills a research gap in the potential effects of long-term air pollution on metabolites in children and young adults. Further, using urinary metabolomics data, we were able to replicate some endogenous compounds and metabolic pathways identified in studies using blood-based metabolomics, such as long-chain fatty acids. The well-defined cohort, which provides extensive information from birth to adult age for the participants, enables sensitivity analyses, enhancing the robustness of our findings. We were also able to perform a post hoc analysis exploring interaction with genetic variants. Another strength of the current study is the large sample size.

The main limitations of this study are that the association between air pollution and exogenous compounds may have been influenced by dietary factors, which we could not explore further. Indoor air pollution and air pollution in occupational settings were not assessed, which might have introduced some misclassification bias, though likely non-differential. The primary results are based on only 260 annotated metabolites, which is significantly less than the 9663 detected features and may result in false negatives for associations of interest. However, the 260 annotated metabolites are high-quality annotations (level 1) and our main results were supported by our supplementary analyses based on the untargeted metabolomics data. Another limitation is that the strong correlations between the air pollutants under study made it difficult to disentangle pollutant-specific associations. The supplementary analyses based on untargeted feature-based datasets have a large amount of missing data; however, our statistical method using a left-censored exponential regression model, suitable for skewed distribution and for a large percentage of missing data, gives robust estimates that are easier to interpret compared with approaches based on transformed values. In addition, the generalizability of the findings is limited by the study population, including a higher proportion of people with allergic symptoms, asthma, and low lung function compared with the general population.

## Conclusions

Both early-life and prior year-long-term air pollution exposures are associated with urine metabolites in childhood and young adulthood. Particularly, our study suggests that children exposed to higher levels of air pollutants during the first year of life or the prior year to biosampling had upregulated caffeine pathway metabolites likely related to perturbations in pathways, indicating possible changes in enzyme activity. Young adults with higher prior-year air pollution exposure had downregulated fatty acid-related metabolic pathways, and our results suggest that this effect was modified by BMI. Given that our study population is generally healthy and living in Stockholm with comparatively clean ambient air, it is noteworthy that metabolic alterations related to air pollution exposure could still be shown.

## Supplementary information

Overview of Supporting information

Appendix A

Tables B.1-B.44

Figs. C.1-C.3

Figs. D.1-D.4

Figs. E.1-E.32

Figs. F.1-F.38

Figs. G.1-G.4

Figs. H.1-H.12

## Data Availability

Additional data are available from the corresponding author on reasonable request.
