# Supplementary material for: Long-term exposure to air pollution and metabolites in children and young adults in a Swedish birth cohort
Source: J Expo Sci Environ Epidemiol. 2025 Oct 3;36(2):251–66. doi: 10.1038/s41370-025-00810-1 (PMC12960235; doi:10.1038/s41370-025-00810-1)
Supplement: Supplementary file 1 — Overview of Supporting information [file 41370_2025_810_MOESM1_ESM.docx]

**Overview of Supporting information**

Appendix A: Supplemental methods description.

Appendix B: Additional descriptive statistics (Tables B.1- B.3), model comparison (Table B.4), results from metabolite-specific analyses and sensitivity analyses (Tables B.4-B.17), stratified analyses (Tables B.18-B.25), genetic variants and air pollution interaction analyses on urine metabolites (Tables B.27-B.44).

Appendix C: A directed acyclic graph (Figure C.1), a mechanistic plot (Figure C.2), and sensitivity analysis (Figure C.3).

Appendix D: Additional volcano plots (Figures D.1-D.4).

Appendices E and F: Density plots of the distribution of each identified metabolite over quartiles of air pollution exposure (Figures E.1-E.32, F.1-F.38).

Appendix G: Additional topology plots of top enriched pathways (Figures G.1-G.4).

Appendix H: Visualization on findings from genetic analyses (Figures H.1-H.12).
