# Supplementary material for: Long-term exposure to air pollution and metabolites in children and young adults in a Swedish birth cohort
Source: J Expo Sci Environ Epidemiol. 2025 Oct 3;36(2):251–66. doi: 10.1038/s41370-025-00810-1 (PMC12960235; doi:10.1038/s41370-025-00810-1)
Supplement: Supplementary file 2 — Appendix A [file 41370_2025_810_MOESM2_ESM.docx]

**Appendix A. Supplemental methods description**

**2.3 Metabolomics data acquisition, processing and metabolite identification.**

LC-HRMS were performed on an Agilent 1290 Infinity II ultra-high performance liquid chromatography (UHPLC) system coupled to 6550 iFunnel quadrupole-time of flight (Q-TOF) mass spectrometer equipped with a dual AJS electrospray ionization source (Agilent Technologies, Santa Clara, CA, USA). Polar metabolites were separated on a SeQuant® ZIC®-p-HILIC (Merck, Darmstadt, Germany) column 100 Å, 100 × 2.1 mm, 5 μm particle size coupled to a guard column SeQuant ZIC-pHILIC, 20 × 2.1 mm, 5 μm particle size and an inline-filter. Mobile phases consisted of (A) ammonium acetate 5 mM with 0.04% ammonium hydroxide in water (pH=9.3) and (B) pure acetonitrile. The fragment or voltage was set at 300 V. The acquisition was performed in DIA mode using a mass range of 40-1200 m/z where full scan high resolution data were acquired at three alternating collision energies (0 eV, 10 eV, and 30 eV).

First, raw data files were converted into centroided mzML using ProteoWizard (Chambers et al. 2012) and loaded into MS-DIAL 4.90 (Tsugawa et al. 2020). Identifications were based on in-house compound libraries containing accurate mass (AM) and retention times (RT) for 622 chemical standards enabling high confidence annotation (level 1) (Sumner et al. 2007). After inspection and curation, peak areas were exported from MS-DIAL for subsequent processing.

**Identified metabolites and remarks**

A total of 260 unique metabolites were annotated in negative and positive ionizations in addition to the three internal standards (tIS). When combining negative and positive modes, if a metabolite was detected in both modes then the mode with better QC CV/intensities was selected. After QCRSC normalization, the CVs of tIS CHES were in negative ionization mode 3.8% in the QC and 17% in the study samples; in positive ionization mode 7.3% and 14% in QC and study samples, respectively. AM and RT differences from the library for the identified metabolites were <10 ppm (except 14 metabolites with high intensities) and <0.9 min, respectively. In the final normalized dataset, all the 260 reported metabolites had the D-Ratios <45% (Broadhurst et al. 2018), and QC CV for most are <30% (except 16 metabolites).

QCRS normalized relative abundances of the 260 unique metabolites in the 1,460 BAMSE urine samples were used in the statistical analyses.

**2.4 Data preparation for statistical analysis**

We treated different metabolites as repeated measures of the overall metabolome of the same individual. To investigate the association between air pollution and the urine metabolome, we initially aggregated annotated metabolites into one outcome variable (referred to as “metabolome”). This was done in the annotated dataset by reshaping metabolite variables from a wide format (i.e. the data spreadsheet contains the metabolites by columns and participants by rows) to one “metabolome” variable in a long format (i.e. the data spreadsheet contains the metabolites by rows and each subject has multiple rows) and creating a metabolome name variable (referred to as “metabolite id”) to identify specific metabolites.

**2.6 Genotyping, quality control analysis, and imputation**

DNA was extracted from blood samples collected from participants at 8 and 16 years of age. Genotyping was carried out in two subsets of individuals, hereon referred to as “Waves”. The Illumina Human 610-quad array (Illumina, Inc.) and the Illumina Infinium Global Screening Array-24 v1.0 BeadChip (Illumina, Inc.) were used in Wave 1 (n=505) and Wave 2 (n=2,387), respectively (Hernandez-Pacheco et al. 2024). Quality control (QC) analyses were performed on subjects and genetic variants separately in each genotyping Wave using PLINK 2.0 (Chang et al. 2015; Purcell et al. 2007). Individuals with call rate (CR) <98%, heterozygosity rates higher or lower than 4 standard deviations of the population mean, sex discordances, and/or evidence of relatedness for at least second-degree relatives (PIHAT ≥0.2) were excluded. Duplicated samples between Waves were retained only in the Wave with the highest CR. Outliers of genetic ancestry identified by the comparison with populations included in the 1,000 Genome Project reference panel (Abecasis et al. 2012) were also excluded. These analyses resulted in 2,636 samples that passed QC procedures (Wave 1, n=463; Wave 2, n=2,173). Analyses were performed combining both Waves (adjusting for wave). Genetic variants with CR <95%, deviations from the Hardy-Weinberg Equilibrium (p<1x10^-6^), and minor allele frequency (MAF) <0.01 were discarded from further analyses. Imputation of genetic variants was independently performed in each Wave using the Positional Burrows-Wheeler Transform (PBWT) software (Durbin 2014), the Haplotype Reference Consortium (HRC) r1.1 reference panel (McCarthy et al. 2016), and the SHAPEIT2 haplotype phasing tool (Delaneau et al. 2008) through the Sanger Imputation Server.

**References**

Abecasis, G.R., Auton, A., Brooks, L.D., DePristo, M.A., Durbin, R.M., Handsaker, R.E., Kang, H.M., Marth, G.T., and McVean, G.A. 2012. An integrated map of genetic variation from 1,092 human genomes, Nature, 491: 56-65.

Broadhurst, D., Goodacre, R., Reinke, S.N., Kuligowski, J., Wilson, I.D., Lewis, M.R., and Dunn, W.B. 2018. Guidelines and considerations for the use of system suitability and quality control samples in mass spectrometry assays applied in untargeted clinical metabolomic studies, Metabolomics, 14: 72.

Chambers, M.C., Maclean, B., Burke, R., Amodei, D., Ruderman, D.L., Neumann, S., Gatto, L., Fischer, B., Pratt, B., Egertson, J., Hoff, K., Kessner, D., Tasman, N., Shulman, N., Frewen, B., Baker, T.A., Brusniak, M.Y., Paulse, C., Creasy, D., Flashner, L., Kani, K., Moulding, C., Seymour, S.L., Nuwaysir, L.M., Lefebvre, B., Kuhlmann, F., Roark, J., Rainer, P., Detlev, S., Hemenway, T., Huhmer, A., Langridge, J., Connolly, B., Chadick, T., Holly, K., Eckels, J., Deutsch, E.W., Moritz, R.L., Katz, J.E., Agus, D.B., MacCoss, M., Tabb, D.L., and Mallick, P. 2012. A cross-platform toolkit for mass spectrometry and proteomics, Nat Biotechnol, 30: 918-20.

Chang, C.C., Chow, C.C., Tellier, L.C., Vattikuti, S., Purcell, S.M., and Lee, J.J. 2015. Second-generation PLINK: rising to the challenge of larger and richer datasets, Gigascience, 4: 7.

Delaneau, O., Coulonges, C., and Zagury, J.F. 2008. Shape-IT: new rapid and accurate algorithm for haplotype inference, BMC Bioinformatics, 9: 540.

Durbin, R. 2014. Efficient haplotype matching and storage using the positional Burrows-Wheeler transform (PBWT), Bioinformatics, 30: 1266-72.

Hernandez-Pacheco, N., Kilanowski, A., Kumar, A., Curtin, J.A., Olvera, N., Kress, S., Bertels, X., Lahousse, L., Bhatta, L., Granell, R., Marí, S., Bilbao, J.R., Sun, Y., Tingskov Pedersen, C.E., Karramass, T., Thiering, E., Dardani, C., Kebede Merid, S., Wang, G., Hallberg, J., Koch, S., Garcia-Aymerich, J., Esplugues, A., Torrent, M., Ibarluzea, J., Lowe, L., Simpson, A., Gehring, U., Vermeulen, R.C.H., Roberts, G., Bergström, A., Vonk, J.M., Felix, J.F., Duijts, L., Bønnelykke, K., Timpson, N., Brusselle, G., Brumpton, B.M., Langhammer, A., Turner, S., Holloway, J.W., Arshad, S.H., Ullah, A., Custovic, A., Cullinan, P., Murray, C.S., van den Berge, M., Kull, I., Schikowski, T., Wedzicha, J.A., Koppelman, G., Faner, R., Agustí, À., Standl, M., and Melén, E. 2024. Exploring the genetics of airflow limitation in lung function across the lifespan - a polygenic risk score study, EClinicalMedicine, 75: 102731.

McCarthy, S., Das, S., Kretzschmar, W., Delaneau, O., Wood, A.R., Teumer, A., Kang, H.M., Fuchsberger, C., Danecek, P., Sharp, K., Luo, Y., Sidore, C., Kwong, A., Timpson, N., Koskinen, S., Vrieze, S., Scott, L.J., Zhang, H., Mahajan, A., Veldink, J., Peters, U., Pato, C., van Duijn, C.M., Gillies, C.E., Gandin, I., Mezzavilla, M., Gilly, A., Cocca, M., Traglia, M., Angius, A., Barrett, J.C., Boomsma, D., Branham, K., Breen, G., Brummett, C.M., Busonero, F., Campbell, H., Chan, A., Chen, S., Chew, E., Collins, F.S., Corbin, L.J., Smith, G.D., Dedoussis, G., Dorr, M., Farmaki, A.E., Ferrucci, L., Forer, L., Fraser, R.M., Gabriel, S., Levy, S., Groop, L., Harrison, T., Hattersley, A., Holmen, O.L., Hveem, K., Kretzler, M., Lee, J.C., McGue, M., Meitinger, T., Melzer, D., Min, J.L., Mohlke, K.L., Vincent, J.B., Nauck, M., Nickerson, D., Palotie, A., Pato, M., Pirastu, N., McInnis, M., Richards, J.B., Sala, C., Salomaa, V., Schlessinger, D., Schoenherr, S., Slagboom, P.E., Small, K., Spector, T., Stambolian, D., Tuke, M., Tuomilehto, J., Van den Berg, L.H., Van Rheenen, W., Volker, U., Wijmenga, C., Toniolo, D., Zeggini, E., Gasparini, P., Sampson, M.G., Wilson, J.F., Frayling, T., de Bakker, P.I., Swertz, M.A., McCarroll, S., Kooperberg, C., Dekker, A., Altshuler, D., Willer, C., Iacono, W., Ripatti, S., Soranzo, N., Walter, K., Swaroop, A., Cucca, F., Anderson, C.A., Myers, R.M., Boehnke, M., McCarthy, M.I., and Durbin, R. 2016. A reference panel of 64,976 haplotypes for genotype imputation, Nat Genet, 48: 1279-83.

Purcell, S., Neale, B., Todd-Brown, K., Thomas, L., Ferreira, M.A., Bender, D., Maller, J., Sklar, P., de Bakker, P.I., Daly, M.J., and Sham, P.C. 2007. PLINK: a tool set for whole-genome association and population-based linkage analyses, Am J Hum Genet, 81: 559-75.

Sumner, L.W., Amberg, A., Barrett, D., Beale, M.H., Beger, R., Daykin, C.A., Fan, T.W., Fiehn, O., Goodacre, R., Griffin, J.L., Hankemeier, T., Hardy, N., Harnly, J., Higashi, R., Kopka, J., Lane, A.N., Lindon, J.C., Marriott, P., Nicholls, A.W., Reily, M.D., Thaden, J.J., and Viant, M.R. 2007. Proposed minimum reporting standards for chemical analysis Chemical Analysis Working Group (CAWG) Metabolomics Standards Initiative (MSI), Metabolomics, 3: 211-21.

Tsugawa, H., Ikeda, K., Takahashi, M., Satoh, A., Mori, Y., Uchino, H., Okahashi, N., Yamada, Y., Tada, I., Bonini, P., Higashi, Y., Okazaki, Y., Zhou, Z., Zhu, Z.J., Koelmel, J., Cajka, T., Fiehn, O., Saito, K., Arita, M., and Arita, M. 2020. A lipidome atlas in MS-DIAL 4, Nat Biotechnol, 38: 1159-63.
