# Supplementary material for: Long-term exposure to air pollution and metabolites in children and young adults in a Swedish birth cohort
Source: J Expo Sci Environ Epidemiol. 2025 Oct 3;36(2):251–66. doi: 10.1038/s41370-025-00810-1 (PMC12960235; doi:10.1038/s41370-025-00810-1)
Supplement: Supplementary file 4 — Figs. C.1-C.3 [file 41370_2025_810_MOESM4_ESM.docx]

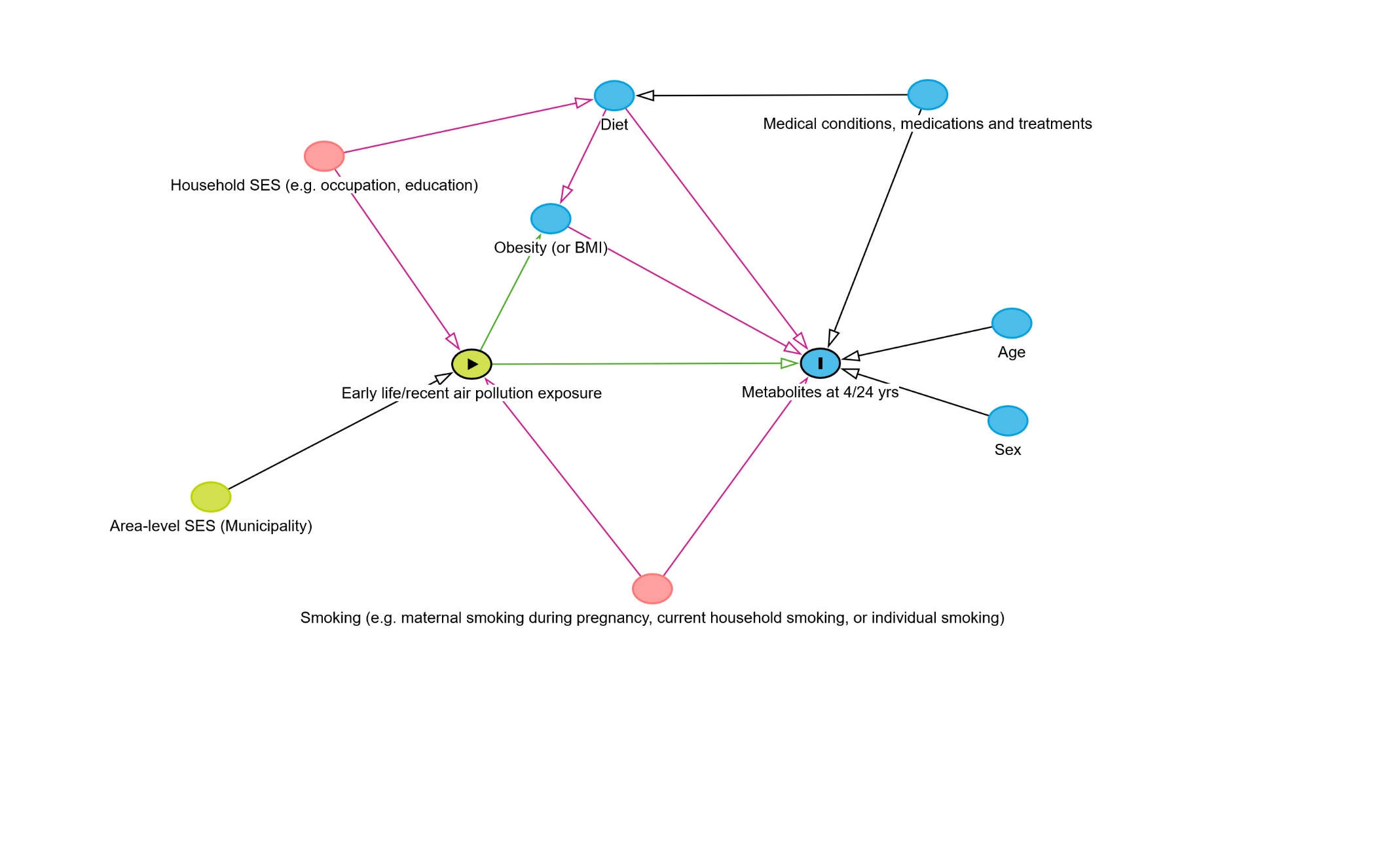


Figure C.1 The hypothesis of the relationship between exposure, outcomes, and covariates: a DAG.


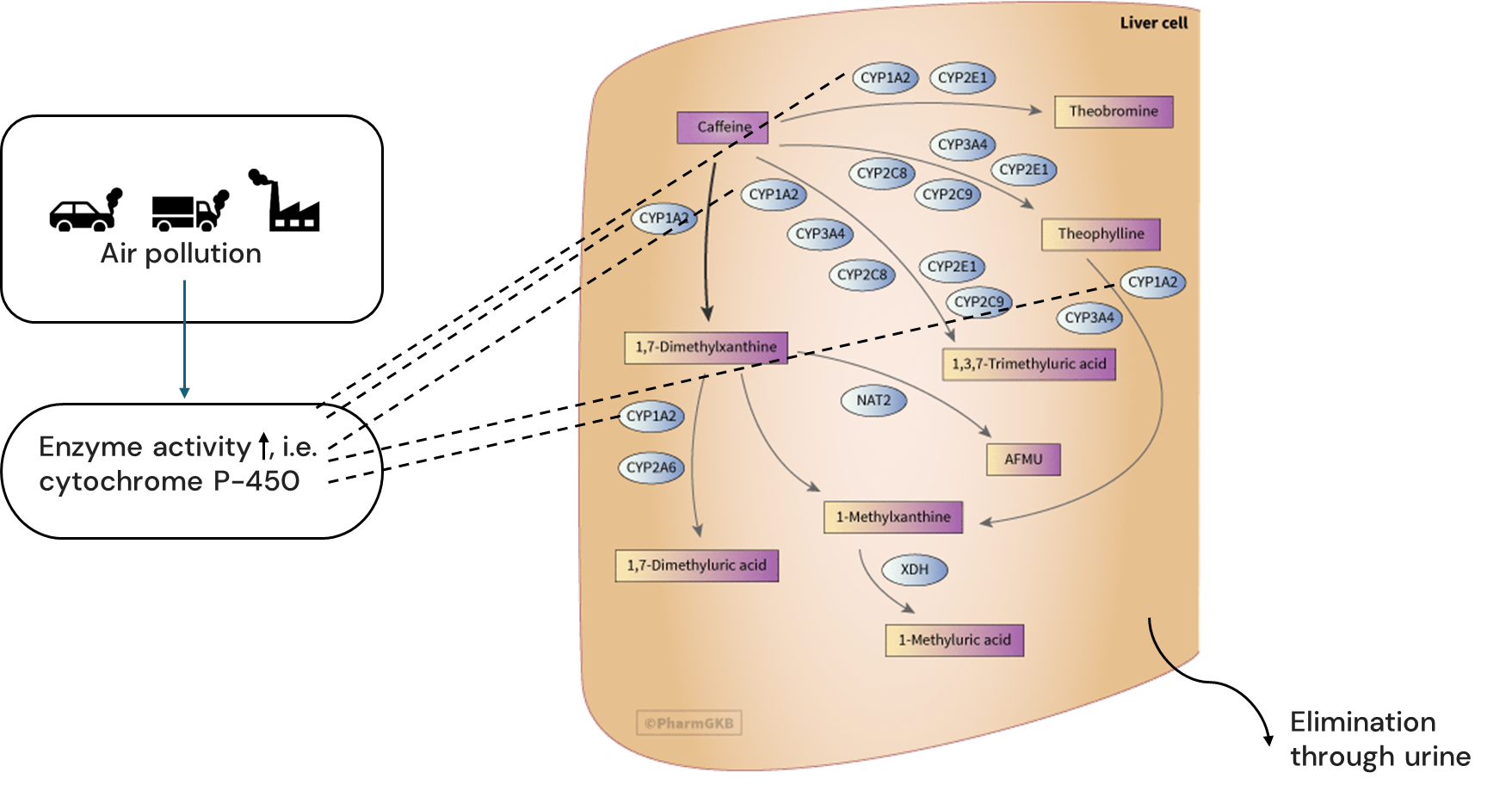


Figure C.2. The hypothesized mechanism through which air pollution affects caffeine metabolism, adapted from a figure by PharmGKB CC BY-SA 4.0 (available online at <http://www.pharmgkb.org/pathway/PA165884757>), based on previous literature (1, 2).


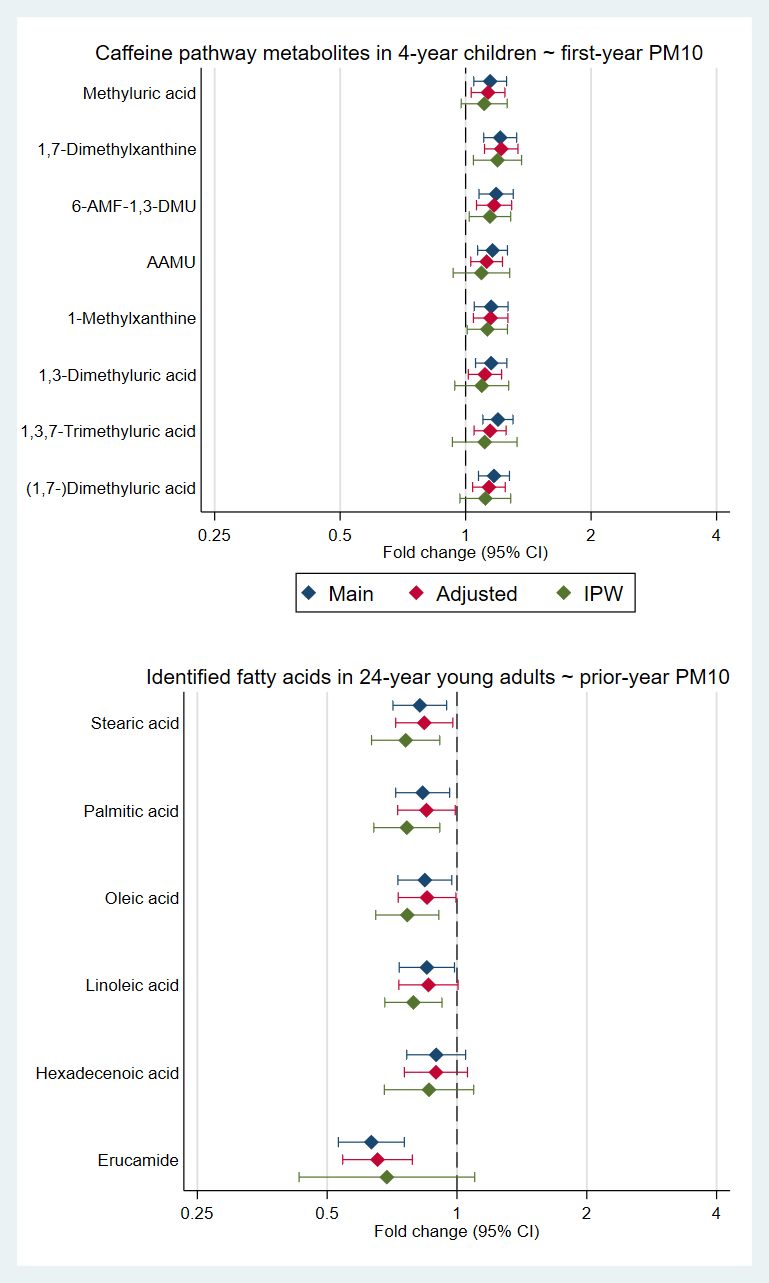


Figure C.3. Sensitivity analyses for main findings (blue) showing models adjusted for selection variables (red) and inverse probability weighting (IPW) for selection variables (green).

Footnote: In 4-year follow-up, the selection variables were asthma and allergic symptoms; In 24-year follow-up, the selection variables were asthma and pre-COPD.

**References**

1. Thorn CF, Aklillu E, McDonagh EM, Klein TE, Altman RB. PharmGKB summary: caffeine pathway. Pharmacogenetics and genomics. 2012;22(5):389-95.

2. Jin X, Chen Y, Xu B, Tian H. Exercise-Mediated Protection against Air Pollution-Induced Immune Damage: Mechanisms, Challenges, and Future Directions. Biology (Basel). 2024;13(4).
