# Supplementary material for: Long-term exposure to air pollution and metabolites in children and young adults in a Swedish birth cohort
Source: J Expo Sci Environ Epidemiol. 2025 Oct 3;36(2):251–66. doi: 10.1038/s41370-025-00810-1 (PMC12960235; doi:10.1038/s41370-025-00810-1)
Supplement: Supplementary file 5 — Figs. D.1-D.4 [file 41370_2025_810_MOESM5_ESM.pdf]

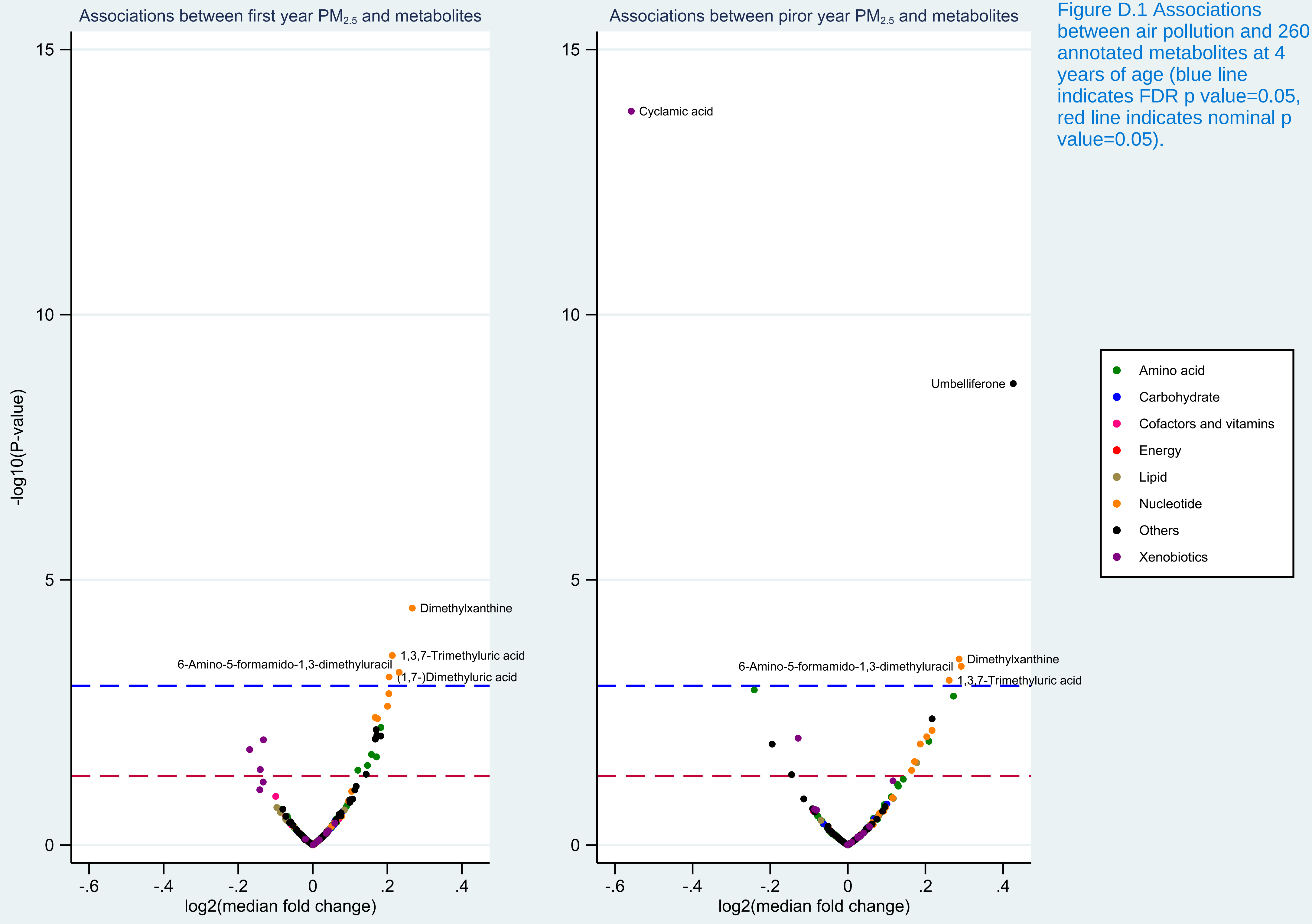

Figure D.1 Associations between air pollution and 260 annotated metabolites at 4 years of age (blue line indicates FDR p value=0.05, red line indicates nominal p value=0.05).

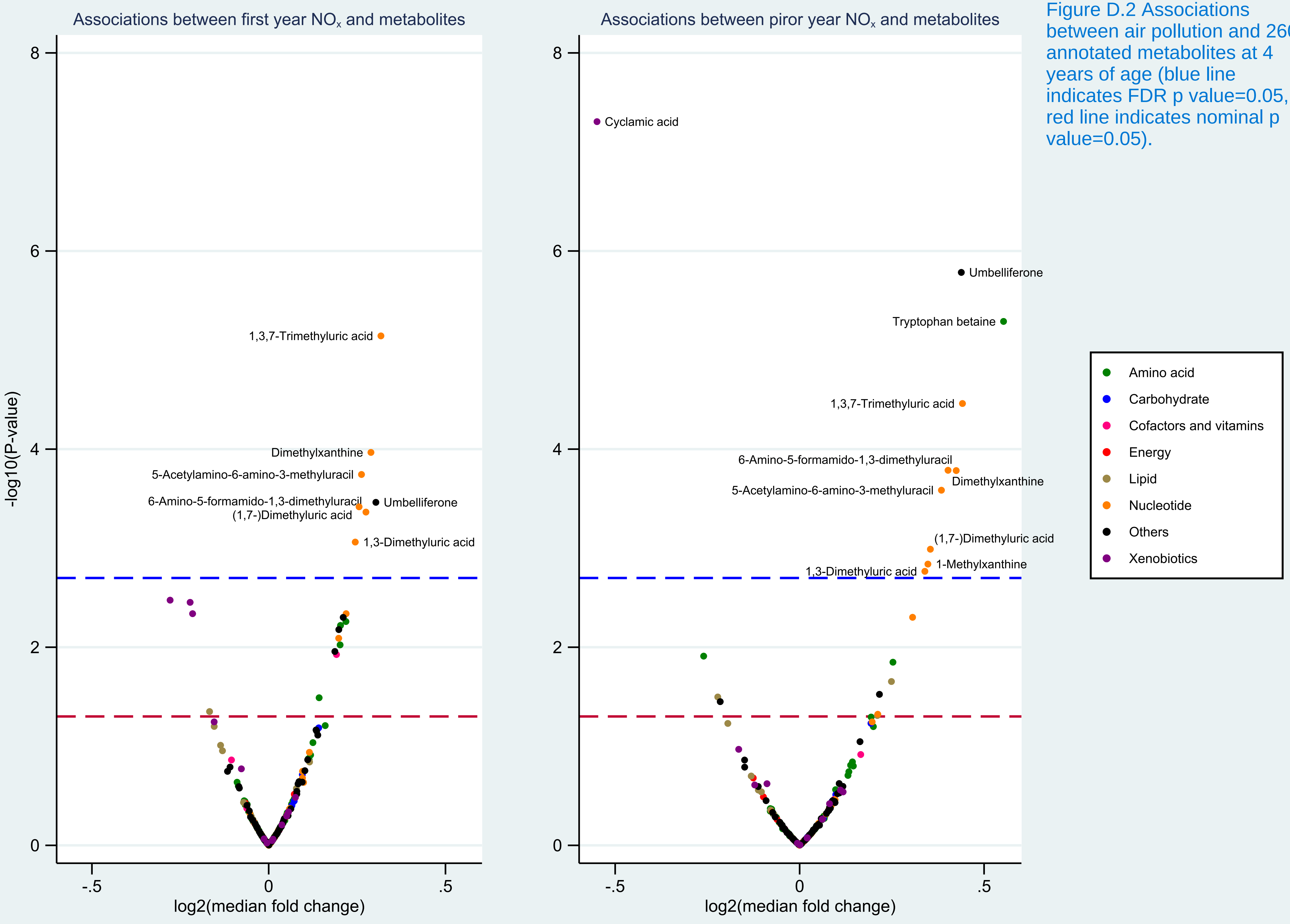

Figure D.2 Associations between air pollution and 260 annotated metabolites at 4 years of age (blue line indicates FDR p value=0.05, red line indicates nominal p value=0.05).

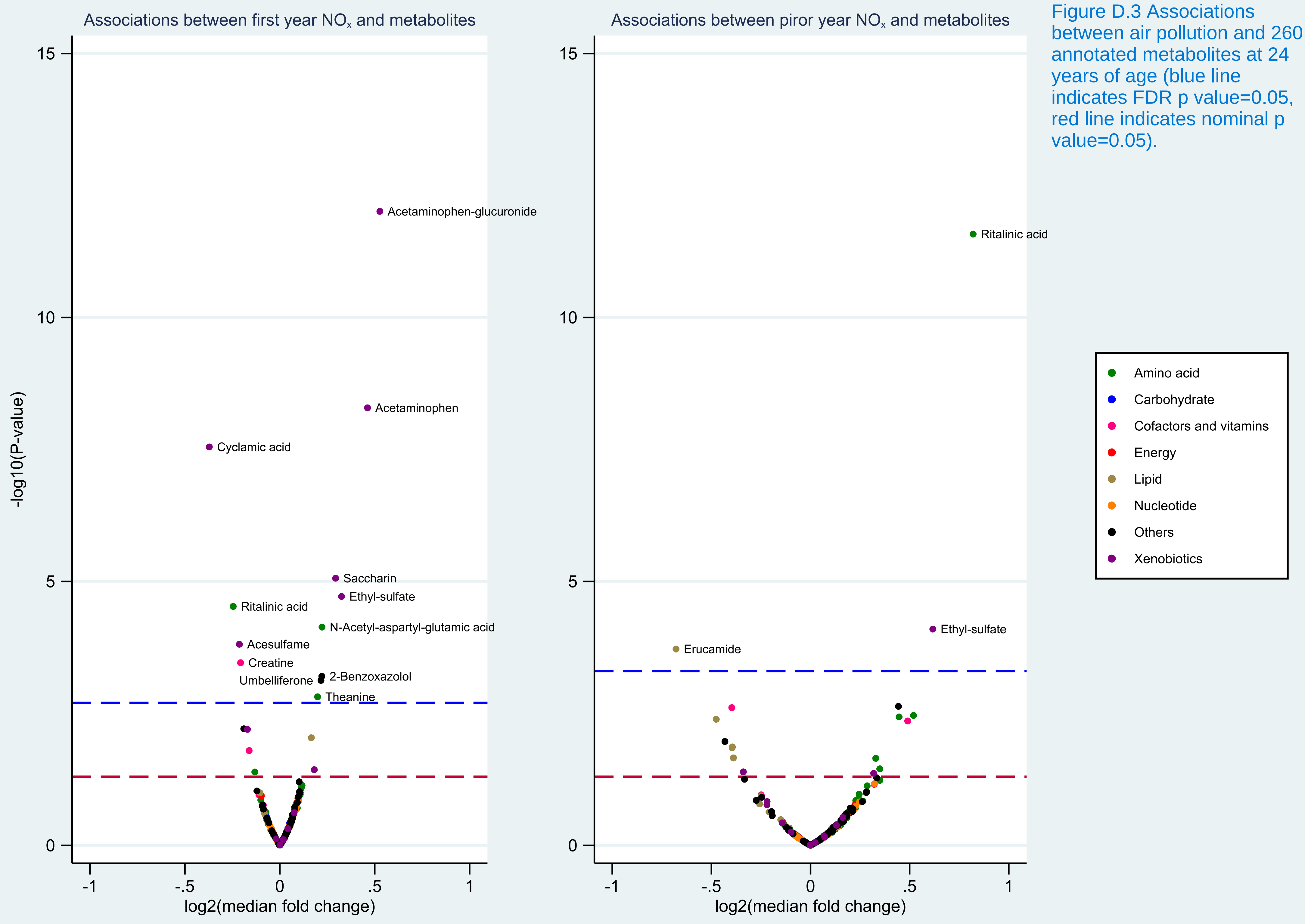

Figure D.3 Associations between air pollution and 260 annotated metabolites at 24 years of age (blue line indicates FDR p value=0.05, red line indicates nominal p value=0.05).

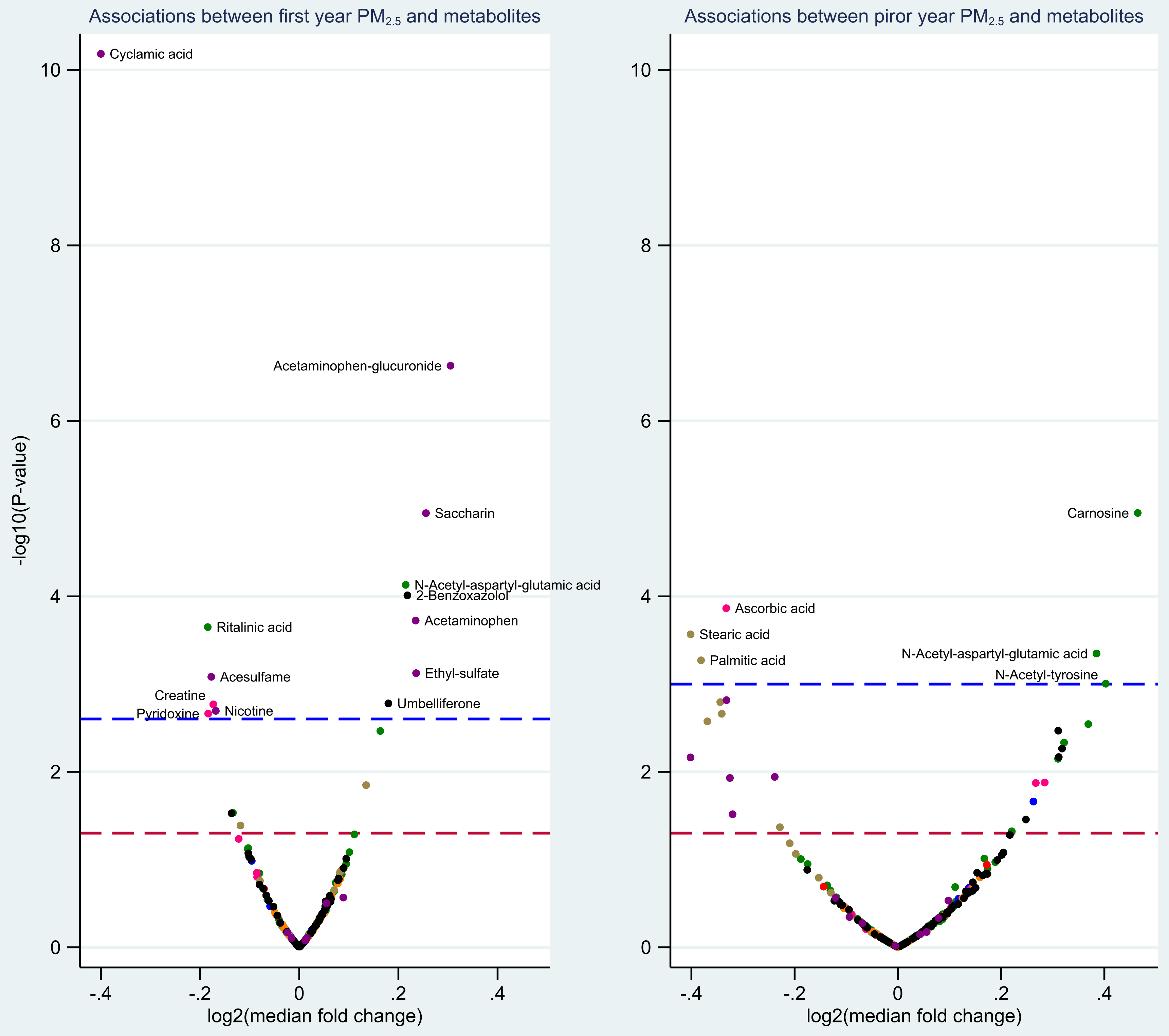

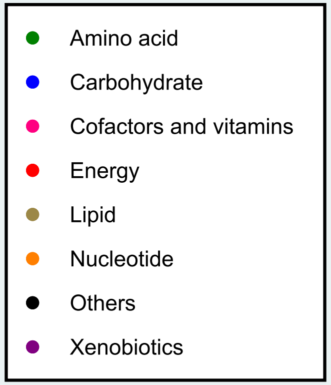

Figure D.4 Associations between air pollution and 260 annotated metabolites at 24 years of age (blue line indicates FDR p value=0.05, red line indicates nominal p value=0.05).
