# Supplementary material for: Long-term exposure to air pollution and metabolites in children and young adults in a Swedish birth cohort
Source: J Expo Sci Environ Epidemiol. 2025 Oct 3;36(2):251–66. doi: 10.1038/s41370-025-00810-1 (PMC12960235; doi:10.1038/s41370-025-00810-1)
Supplement: Supplementary file 6 — Figs. E.1-E.32 [file 41370_2025_810_MOESM6_ESM.docx]

Figure E.1. Bivariate visual assessment of association between air pollution (NOx during the first year of life as an example) and 4-year significant metabolites (1,3,7-Trimethyluric acid, FDR p <0.05). Observations over 99th percentile were replaced with the value of the 99th percentile.

Figure E.2. Bivariate visual assessment of association between air pollution (NOx during the first year of life as an example) and 4-year significant metabolites (Dimethylxanthine, FDR p <0.05). Observations over 99th percentile were replaced with the value of the 99th percentile.

Figure E.3. Bivariate visual assessment of association between air pollution (NOx during the first year of life as an example) and 4-year significant metabolites (6-Amino-5-formamido-1,3-dimethyluracil, FDR p <0.05). Observations over 99th percentile were replaced with the value of the 99th percentile.

Figure E.4. Bivariate visual assessment of association between air pollution (NOx during the prior year of life as an example) and 4-year significant metabolites (1-Methylxanthine, FDR p <0.05). Observations over 99th percentile were replaced with the value of the 99th percentile.

Figure E.5. Bivariate visual assessment of association between air pollution (NOx during the first year of life as an example) and 4-year significant metabolites ((1,7-)Dimethyluric acid, FDR p <0.05). Observations over 99th percentile were replaced with the value of the 99th percentile.

Figure E.6. Bivariate visual assessment of association between air pollution (NOx during the first year of life as an example) and 4-year significant metabolites (6-Amino-5-formamido-1,3-dimethyluracil, FDR p <0.05). Observations over 99th percentile were replaced with the value of the 99th percentile.

Figure E.7. Bivariate visual assessment of association between air pollution (NOx during the first year of life as an example) and 4-year significant metabolites (1,3-Dimethyluric acid, FDR p <0.05). Observations over 99th percentile were replaced with the value of the 99th percentile.

Figure E.8. Bivariate visual assessment of association between air pollution (NOx during the first year of life as an example) and 4-year nominally significant metabolites (Methyluric acid, p <0.05). Observations over 99th percentile were replaced with the value of the 99th percentile.

Figure E.9. Bivariate visual assessment of association between air pollution (NOx during the prior year of life as an example) and 4-year nominally significant metabolites (2-O-Methylinosine, p <0.05). Observations over 99th percentile were replaced with the value of the 99th percentile.

Figure E.10. Bivariate visual assessment of association between air pollution (NOx during the prior year of life as an example) and 4-year significant metabolites (Tryptophan betaine, FDR p <0.05). Observations over 99th percentile were replaced with the value of the 99th percentile.

Figure E.11. Bivariate visual assessment of association between air pollution (NOx during the first year of life as an example) and 4-year nominally significant metabolites (Anserine, p <0.05). Observations over 99th percentile were replaced with the value of the 99th percentile.

Figure E.12. Bivariate visual assessment of association between air pollution (NOx during the first year of life as an example) and 4-year nominally significant metabolites (Succinylacetone, p <0.05). Observations over 99th percentile were replaced with the value of the 99th percentile.

Figure E.13. Bivariate visual assessment of association between air pollution (NOx during the prior year of life as an example) and 4-year nominally significant metabolites (cis-Urocanic acid, p <0.05). Observations over 99th percentile were replaced with the value of the 99th percentile.

Figure E.14. Bivariate visual assessment of association between air pollution (NOx during the first year of life as an example) and 4-year nominally significant metabolites (Furosine, p <0.05). Observations over 99th percentile were replaced with the value of the 99th percentile.

Figure E.15. Bivariate visual assessment of association between air pollution (NOx during the first year of life as an example) and 4-year nominally significant metabolites (N-Methyl-proline, p <0.05). Observations over 99th percentile were replaced with the value of the 99th percentile.

Figure E.16. Bivariate visual assessment of association between air pollution (PM2.5 during the prior year of life as an example) and 4-year nominally significant metabolites (Methyl-histidine, p <0.05). Observations over 99th percentile were replaced with the value of the 99th percentile.

Figure E.17. Bivariate visual assessment of association between air pollution (NOx during the prior year of life as an example) and 4-year nominally significant metabolites (Azelaic acid, p <0.05). Observations over 99th percentile were replaced with the value of the 99th percentile.

Figure E.18. Bivariate visual assessment of association between air pollution (NOx during the prior year of life as an example) and 4-year nominally significant metabolites (Suberic acid, p <0.05). Observations over 99th percentile were replaced with the value of the 99th percentile.

Figure E.19. Bivariate visual assessment of association between air pollution (NOx during the first year of life as an example) and 4-year nominally significant metabolites (Stearic acid, p <0.05). Observations over 99th percentile were replaced with the value of the 99th percentile.

Figure E.20. Bivariate visual assessment of association between air pollution (NOx during the first year of life as an example) and 4-year nominally significant metabolites (Panthenol, p <0.05). Observations over 99th percentile were replaced with the value of the 99th percentile.

Figure E.21. Bivariate visual assessment of association between air pollution (NOx during the first year of life as an example) and 4-year significant metabolites (Umbelliferone, FDR p <0.05). Observations over 99th percentile were replaced with the value of the 99th percentile.

Figure E.22. Bivariate visual assessment of association between air pollution (NOx during the first year of life as an example) and 4-year nominally significant metabolites (4-Hydroxycoumarin, p <0.05). Observations over 99th percentile were replaced with the value of the 99th percentile.

Figure E.23. Bivariate visual assessment of association between air pollution (PM10 during the first year of life as an example) and 4-year nominally significant metabolites (Quinic acid, p <0.05). Observations over 99th percentile were replaced with the value of the 99th percentile.

Figure E.24. Bivariate visual assessment of association between air pollution (PM10 during the first year of life as an example) and 4-year nominally significant metabolites (Ferulic acid 4-sulfate, p <0.05). Observations over 99th percentile were replaced with the value of the 99th percentile.

Figure E.25. Bivariate visual assessment of association between air pollution (PM10 during the first year of life as an example) and 4-year nominally significant metabolites (2-Benzoxazolol, p <0.05). Observations over 99th percentile were replaced with the value of the 99th percentile.

Figure E.26. Bivariate visual assessment of association between air pollution (PM10 during the first year of life as an example) and 4-year nominally significant metabolites (Adipic Acid, p <0.05). Observations over 99th percentile were replaced with the value of the 99th percentile.

Figure E.27. Bivariate visual assessment of association between air pollution (PM2.5 during the prior year of life as an example) and 4-year nominally significant metabolites (Trimethylamine N-Oxide, p <0.05). Observations over 99th percentile were replaced with the value of the 99th percentile.

Figure E.28. Bivariate visual assessment of association between air pollution (NOx during the prior year of life as an example) and 4-year nominally significant metabolites (2-Piperidone, p <0.05). Observations over 99th percentile were replaced with the value of the 99th percentile.

Figure E.29. Xenobiotics: Bivariate visual assessment of association between air pollution (NOx during the prior year of life as an example) and 4-year significant metabolites (Cyclamic acid, FDR p <0.05). Observations over 99th percentile were replaced with the value of the 99th percentile.

Figure E.30. Xenobiotics: Bivariate visual assessment of association between air pollution (NOx during the first year of life as an example) and 4-year nominally significant metabolites (Saccharin, p <0.05). Observations over 99th percentile were replaced with the value of the 99th percentile.

Figure E.31. Xenobiotics: Bivariate visual assessment of association between air pollution (PM2.5 during the first year of life as an example) and 4-year nominally significant metabolites (Acesulfame, p <0.05). Observations over 99th percentile were replaced with the value of the 99th percentile.

Figure E.32. Xenobiotics: Bivariate visual assessment of association between air pollution (NOx during the first year of life as an example) and 4-year nominally significant metabolites (Acetaminophen, p <0.05). Observations over 99th percentile were replaced with the value of the 99th percentile.
