# Supplementary material for: Long-term exposure to air pollution and metabolites in children and young adults in a Swedish birth cohort
Source: J Expo Sci Environ Epidemiol. 2025 Oct 3;36(2):251–66. doi: 10.1038/s41370-025-00810-1 (PMC12960235; doi:10.1038/s41370-025-00810-1)
Supplement: Supplementary file 7 — Figs. F.1-F.38 [file 41370_2025_810_MOESM7_ESM.docx]

Figure F.1. Bivariate visual assessment of association between air pollution (PM2.5 during the prior year of life as an example) and 24-year significant metabolites (Stearic acid, FDR p <0.05). Observations over 99th percentile were replaced with the value of the 99th percentile.

Figure F.2. Bivariate visual assessment of association between air pollution (PM2.5 during the prior year of life as an example) and 24-year significant metabolites (Palmitic acid, FDR p <0.05). Observations over 99th percentile were replaced with the value of the 99th percentile.

Figure F.3. Bivariate visual assessment of association between air pollution (NOx during the prior year of life as an example) and 24-year nominally significant metabolites (Oleic acid, p <0.05). Observations over 99th percentile were replaced with the value of the 99th percentile.

Figure F.4. Bivariate visual assessment of association between air pollution (NOx during the prior year of life as an example) and 24-year nominally significant metabolites (Linoleic acid, p <0.05). Observations over 99th percentile were replaced with the value of the 99th percentile.

Figure F.5. Bivariate visual assessment of association between air pollution (NOx during the prior year of life as an example) and 24-year significant metabolites (Erucamide, FDR p <0.05). Observations over 99th percentile were replaced with the value of the 99th percentile.

Figure F.6. Bivariate visual assessment of association between air pollution (PM2.5 during the prior year of life as an example) and 24-year nominally significant metabolites (Hexadecenoic acid, p <0.05). Observations over 99th percentile were replaced with the value of the 99th percentile.

Figure F.7. Bivariate visual assessment of association between air pollution (NOx during the first year of life as an example) and 24-year nominally significant metabolites (Azelaic acid, p <0.05). Observations over 99th percentile were replaced with the value of the 99th percentile.

Figure F.8. Bivariate visual assessment of association between air pollution (NOx during the prior year of life as an example) and 24-year significant metabolites (Carnosine, FDR p <0.05). Observations over 99th percentile were replaced with the value of the 99th percentile.

Figure F.9. Bivariate visual assessment of association between air pollution (NOx during the first year of life as an example) and 24-year significant metabolites (N-Acetyl-aspartyl-glutamic acid, FDR p <0.05). Observations over 99th percentile were replaced with the value of the 99th percentile.

Figure F.10. Bivariate visual assessment of association between air pollution (PM2.5 during the prior year of life as an example) and 24-year significant metabolites (N-Acetyl-tyrosine, p <0.05). Observations over 99th percentile were replaced with the value of the 99th percentile.

Figure F.11. Bivariate visual assessment of association between air pollution (PM2.5 during the prior year of life as an example) and 24-year nominally significant metabolites (Alliin, p <0.05). Observations over 99th percentile were replaced with the value of the 99th percentile.

Figure F.12. Bivariate visual assessment of association between air pollution (PM2.5 during the prior year of life as an example) and 24-year nominally significant metabolites (3-Hydroxyanthranilic acid, p <0.05). Observations over 99th percentile were replaced with the value of the 99th percentile.

Figure F.13. Bivariate visual assessment of association between air pollution (PM2.5 during the prior year of life as an example) and 24-year nominally significant metabolites (Anserine, p <0.05). Observations over 99th percentile were replaced with the value of the 99th percentile.

Figure F.14. Bivariate visual assessment of association between air pollution (PM2.5 during the prior year of life as an example) and 24-year nominally significant metabolites (N-Cinnamoylglycine, p <0.05). Observations over 99th percentile were replaced with the value of the 99th percentile.

Figure F.15. Bivariate visual assessment of association between air pollution (NOx during the prior year of life as an example) and 24-year nominally significant metabolites (Betonicine, p <0.05). Observations over 99th percentile were replaced with the value of the 99th percentile.

Figure F.16. Bivariate visual assessment of association between air pollution (NOx during the first year of life as an example) and 24-year nominally significant metabolites (Taurine, p <0.05). Observations over 99th percentile were replaced with the value of the 99th percentile.

Figure F.17. Bivariate visual assessment of association between air pollution (NOx during the first year of life as an example) and 24-year significant metabolites (Ritalinic acid, FDR p <0.05). Observations over 99th percentile were replaced with the value of the 99th percentile.

Figure F.18. Bivariate visual assessment of association between air pollution (NOx during the first year of life as an example) and 24-year significant metabolites (Theanine, FDR p <0.05). Observations over 99th percentile were replaced with the value of the 99th percentile.

Figure F.19. Bivariate visual assessment of association between air pollution (PM2.5 during the prior year of life as an example) and 24-year nominally significant metabolites (Hexitol, p <0.05). Observations over 99th percentile were replaced with the value of the 99th percentile.

Figure F.20. Bivariate visual assessment of association between air pollution (PM2.5 during the prior year of life as an example) and 24-year significant metabolites (Ascorbic acid, FDR p <0.05). Observations over 99th percentile were replaced with the value of the 99th percentile.

Figure F.21. Bivariate visual assessment of association between air pollution (PM2.5 during the prior year of life as an example) and 24-year nominally significant metabolites (Nicotinic acid, p <0.05). Observations over 99th percentile were replaced with the value of the 99th percentile.

Figure F.22. Bivariate visual assessment of association between air pollution (PM2.5 during the first year of life as an example) and 24-year significant metabolites (Pyridoxine, FDR p <0.05). Observations over 99th percentile were replaced with the value of the 99th percentile.

Figure F.23. Bivariate visual assessment of association between air pollution (PM2.5 during the first year of life as an example) and 24-year significant metabolites (Creatine, FDR p <0.05). Observations over 99th percentile were replaced with the value of the 99th percentile.

Figure F.24. Bivariate visual assessment of association between air pollution (PM10 during the first year of life as an example) and 24-year significant metabolites (Umbelliferone, FDR p <0.05). Observations over 99th percentile were replaced with the value of the 99th percentile.

Figure F.25. Bivariate visual assessment of association between air pollution (PM10 during the first year of life as an example) and 24-year significant metabolites (2-Benzoxazolol, FDR p <0.05). Observations over 99th percentile were replaced with the value of the 99th percentile.

Figure F.26. Bivariate visual assessment of association between air pollution (PM10 during the first year of life as an example) and 24-year nominally significant metabolites (Homogentisic acid, p <0.05). Observations over 99th percentile were replaced with the value of the 99th percentile.

Figure F.27. Bivariate visual assessment of association between air pollution (PM2.5 during the recent year of life as an example) and 24-year nominally significant metabolites (Tartaric acid, p <0.05). Observations over 99th percentile were replaced with the value of the 99th percentile.

Figure F.28. Bivariate visual assessment of association between air pollution (PM2.5 during the recent year of life as an example) and 24-year nominally significant metabolites (3-Hydroxypyridine, p <0.05). Observations over 99th percentile were replaced with the value of the 99th percentile.

Figure F.29. Bivariate visual assessment of association between air pollution (PM2.5 during the recent year of life as an example) and 24-year nominally significant metabolites (2-Hydroxypyridine, p <0.05). Observations over 99th percentile were replaced with the value of the 99th percentile.

Figure F.30. Bivariate visual assessment of association between air pollution (PM10 during the recent year of life as an example) and 24-year nominally significant metabolites (Lactic acid, p <0.05). Observations over 99th percentile were replaced with the value of the 99th percentile.

Figure F.31. Xenobiotics: Bivariate visual assessment of association between air pollution (NOx during the first year of life as an example) and 24-year significant metabolites (Saccharin, FDR p <0.05). Observations over 99th percentile were replaced with the value of the 99th percentile.

Figure F.32. Xenobiotics: Bivariate visual assessment of association between air pollution (NOx during the first year of life as an example) and 24-year significant metabolites (Acetaminophen-glucuronide, FDR p <0.05). Observations over 99th percentile were replaced with the value of the 99th percentile.

Figure F.33. Xenobiotics: Bivariate visual assessment of association between air pollution (NOx during the first year of life as an example) and 24-year nominally significant metabolites (Ethyl-glucuronide, p <0.05). Observations over 99th percentile were replaced with the value of the 99th percentile.

Figure F.34. Xenobiotics: Bivariate visual assessment of association between air pollution (PM2.5 during the first year of life as an example) and 24-year significant metabolites (Nicotine, FDR p <0.05). Observations over 99th percentile were replaced with the value of the 99th percentile.

Figure F.35. Xenobiotics: Bivariate visual assessment of association between air pollution (NOx during the first year of life as an example) and 24-year significant metabolites (Acetaminophen, FDR p <0.05). Observations over 99th percentile were replaced with the value of the 99th percentile.

Figure F.36. Xenobiotics: Bivariate visual assessment of association between air pollution (NOx during the first year of life as an example) and 24-year significant metabolites (Acesulfame, FDR p <0.05). Observations over 99th percentile were replaced with the value of the 99th percentile.

Figure F.37. Xenobiotics: Bivariate visual assessment of association between air pollution (NOx during the first year of life as an example) and 24-year significant metabolites (Ethyl-sulfate, FDR p <0.05). Observations over 99th percentile were replaced with the value of the 99th percentile.

Figure F.38. Xenobiotics: Bivariate visual assessment of association between air pollution (NOx during the first year of life as an example) and 24-year significant metabolites (Cyclamic acid, FDR p <0.05). Observations over 99th percentile were replaced with the value of the 99th percentile.
