# Supplementary material for: Long-term exposure to air pollution and metabolites in children and young adults in a Swedish birth cohort
Source: J Expo Sci Environ Epidemiol. 2025 Oct 3;36(2):251–66. doi: 10.1038/s41370-025-00810-1 (PMC12960235; doi:10.1038/s41370-025-00810-1)
Supplement: Supplementary file 8 — Figs. G.1-G.4 [file 41370_2025_810_MOESM8_ESM.docx]

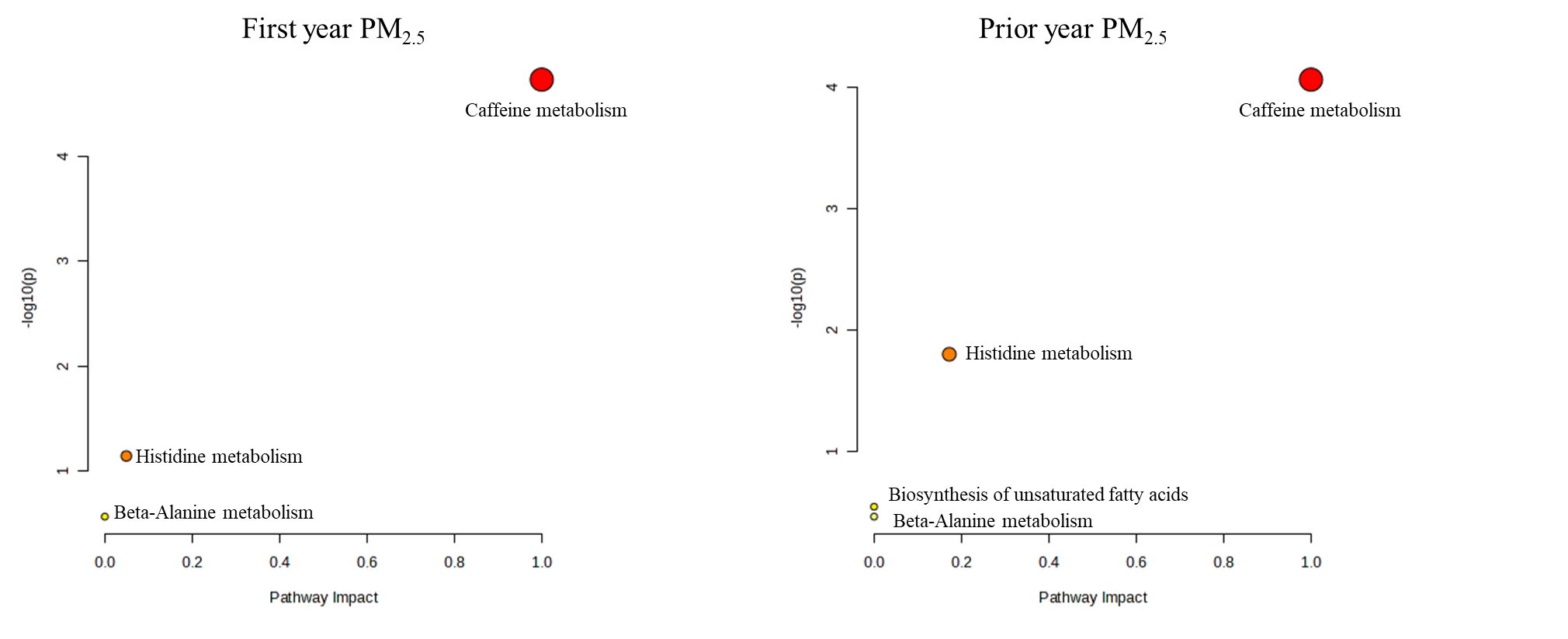


**Figure G.1. Topology plots of top enriched pathways at age 4, associated with air pollution (PM_2.5_).**

**
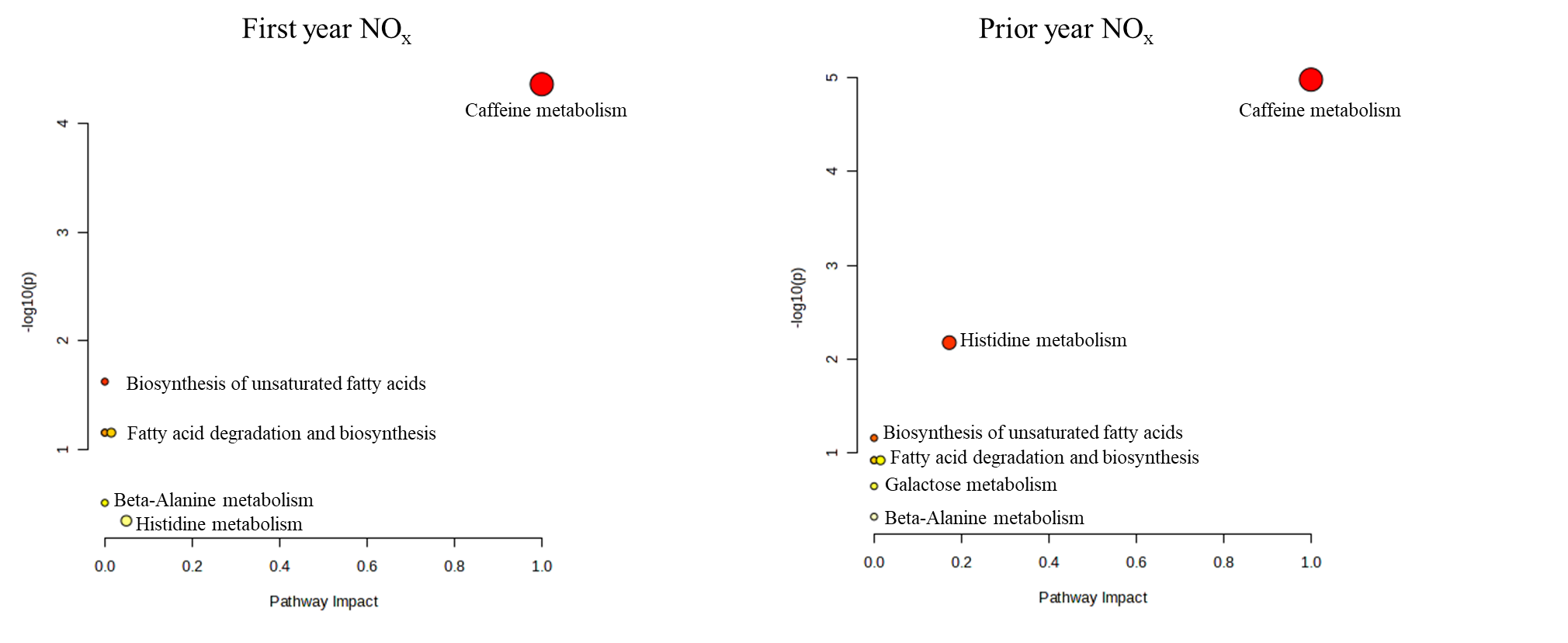
**

**Figure G.2. Topology plots of top enriched pathways at age 4, associated with air pollution (NO_x_).**

**
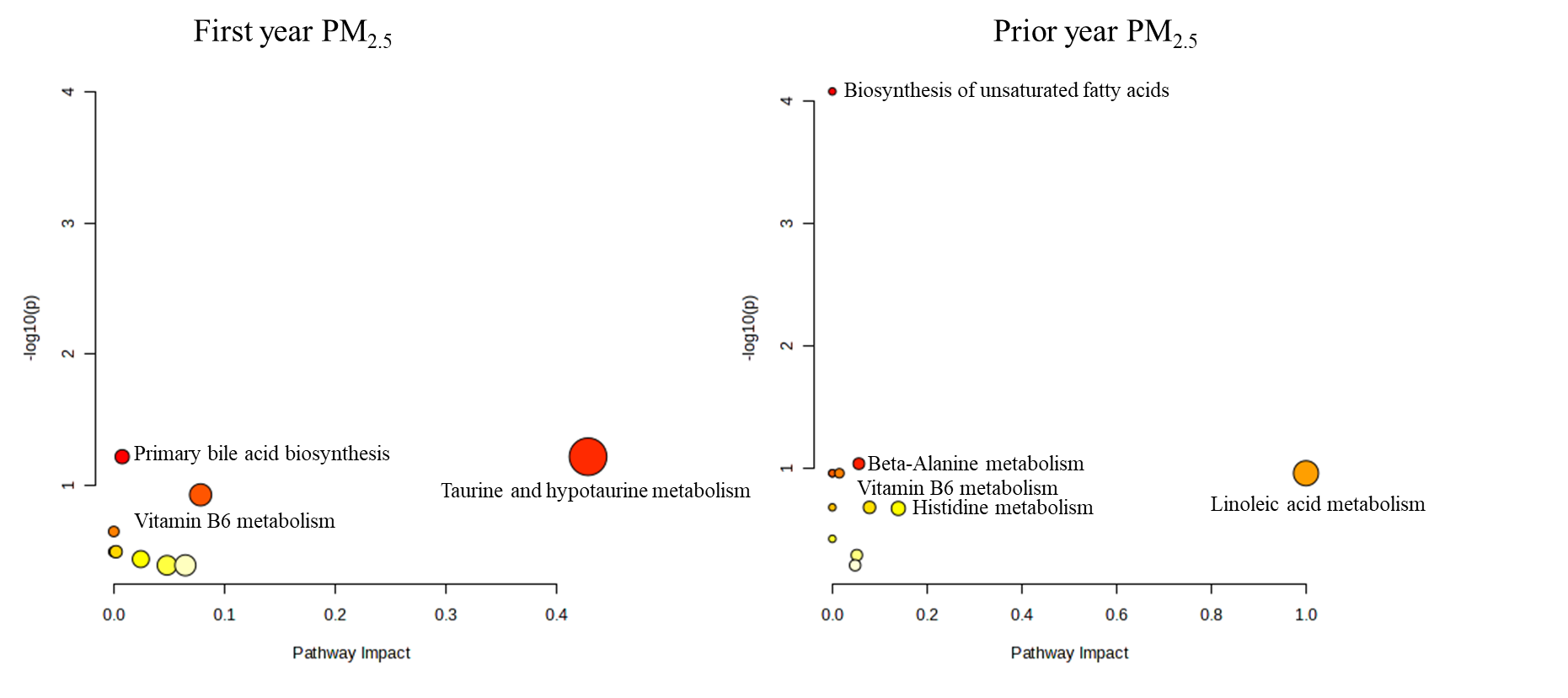
**

**Figure G.3. Topology plots of top enriched pathways at age 24, associated with air pollution (PM_2.5_).**

**
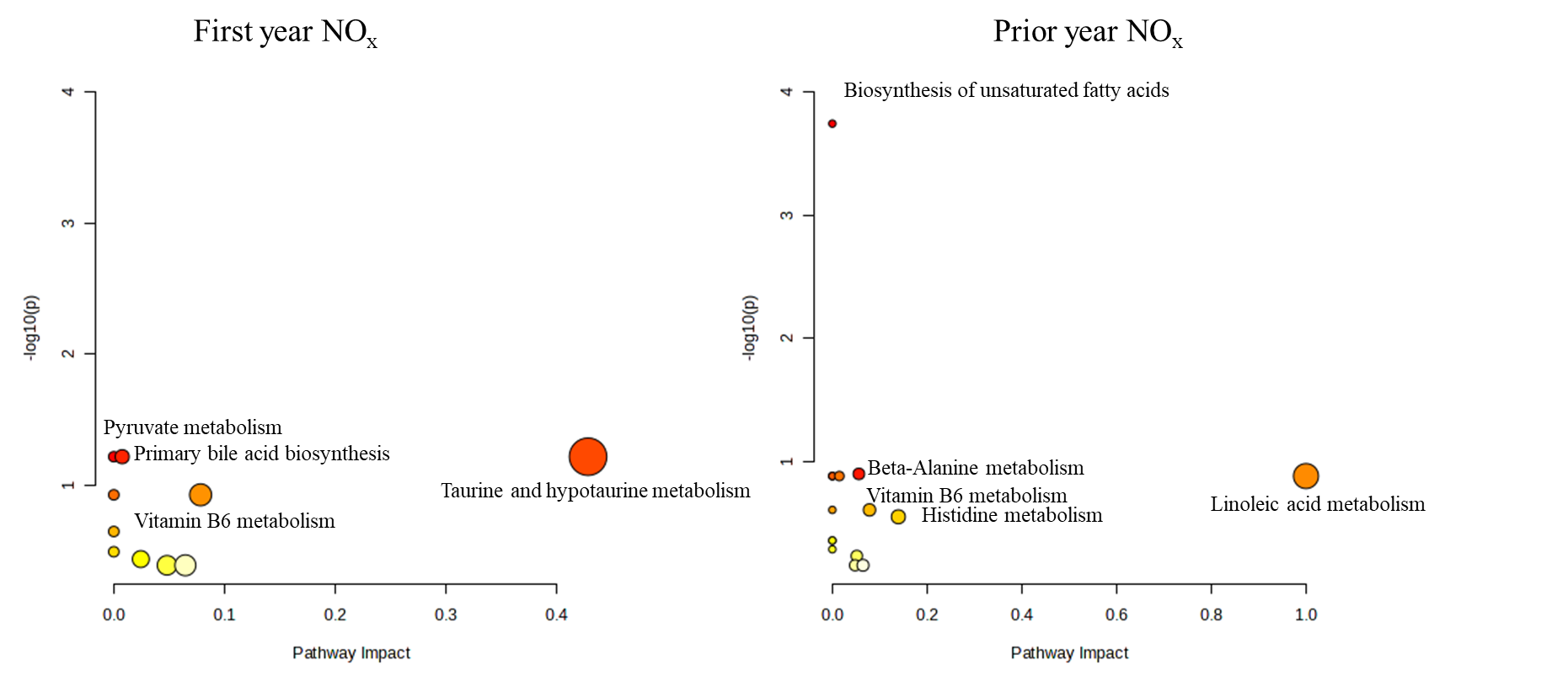
**

**Figure G.4. Topology plots of top enriched pathways at age 24, associated with air pollution (NO_x_).**
