# Supplementary material for: Long-term exposure to air pollution and metabolites in children and young adults in a Swedish birth cohort
Source: J Expo Sci Environ Epidemiol. 2025 Oct 3;36(2):251–66. doi: 10.1038/s41370-025-00810-1 (PMC12960235; doi:10.1038/s41370-025-00810-1)
Supplement: Supplementary file 9 — Figs. H.1-H.12 [file 41370_2025_810_MOESM9_ESM.docx]

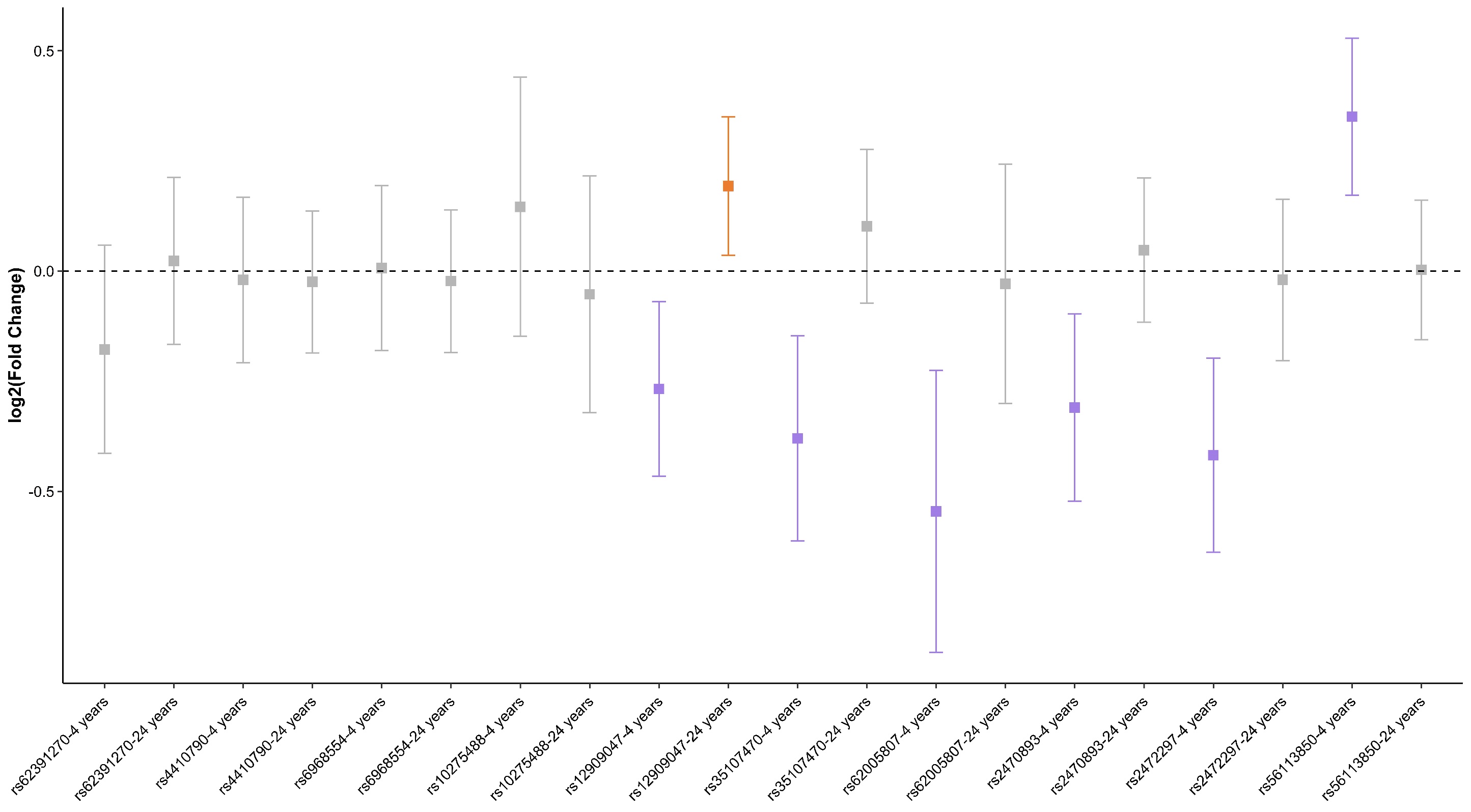


**Figure H.1. Association results of caffeine-metabolism associated genetic variants with 5-Acetylamino-6-amino-3-methyluracil in children and young adults.** Plot comparing the results of the exponential regression model evaluating the association of each SNP with metabolites measured in urine at 4 and 24 years of age. Analyses were carried out in both genotyping Waves of BAMSE (n= 492). The effect size of the association of each SNP (*x*-axis) with the metabolite levels is shown in terms of log_2_(Fold change) (*y*-axis) by boxes. Purple and orange boxes show the association effect estimate with metabolite levels measured in urine samples collected at the 4-year follow-up and 24-year follow-up, respectively (significant associations (p<0.05). The gray boxes represent non-significant results (p>0.05).


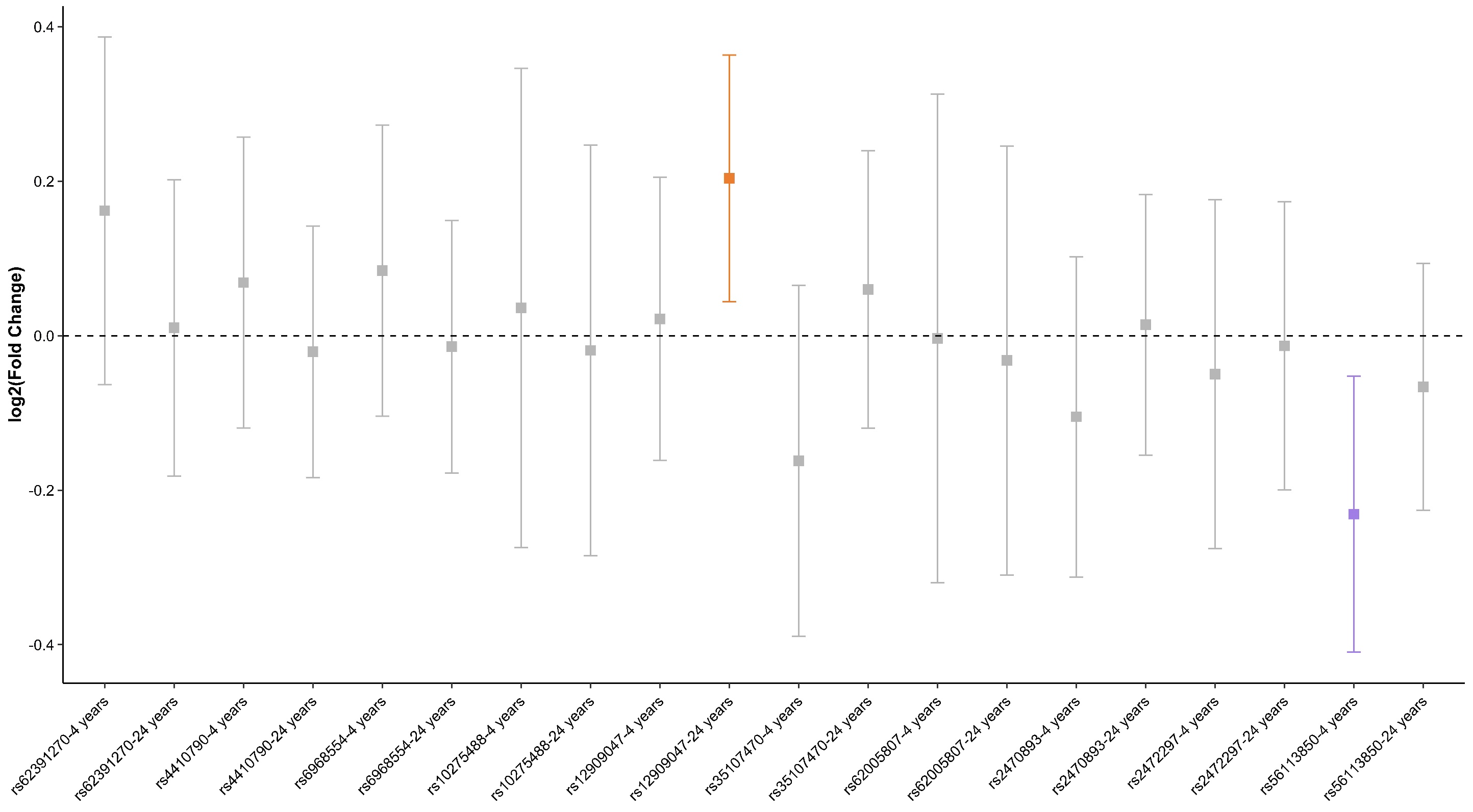


**Figure H.2. Association results of caffeine-metabolism associated genetic variants with 6-Amino-5-formamido-1,3-dimethyluracil in children and young adults.** Plot comparing the results of the exponential regression model evaluating the association of each SNP with metabolites measured in urine at 4 and 24 years of age. Analyses were carried out in both genotyping Waves of BAMSE (n= 492). The effect size of the association of each SNP (*x*-axis) with the metabolite levels is shown in terms of log_2_(Fold change) (*y*-axis) by boxes. Purple and orange boxes show the association effect estimate with metabolite levels measured in urine samples collected at the 4-year follow-up and 24-year follow-up, respectively (significant associations (p<0.05). The gray boxes represent non-significant results (p>0.05).


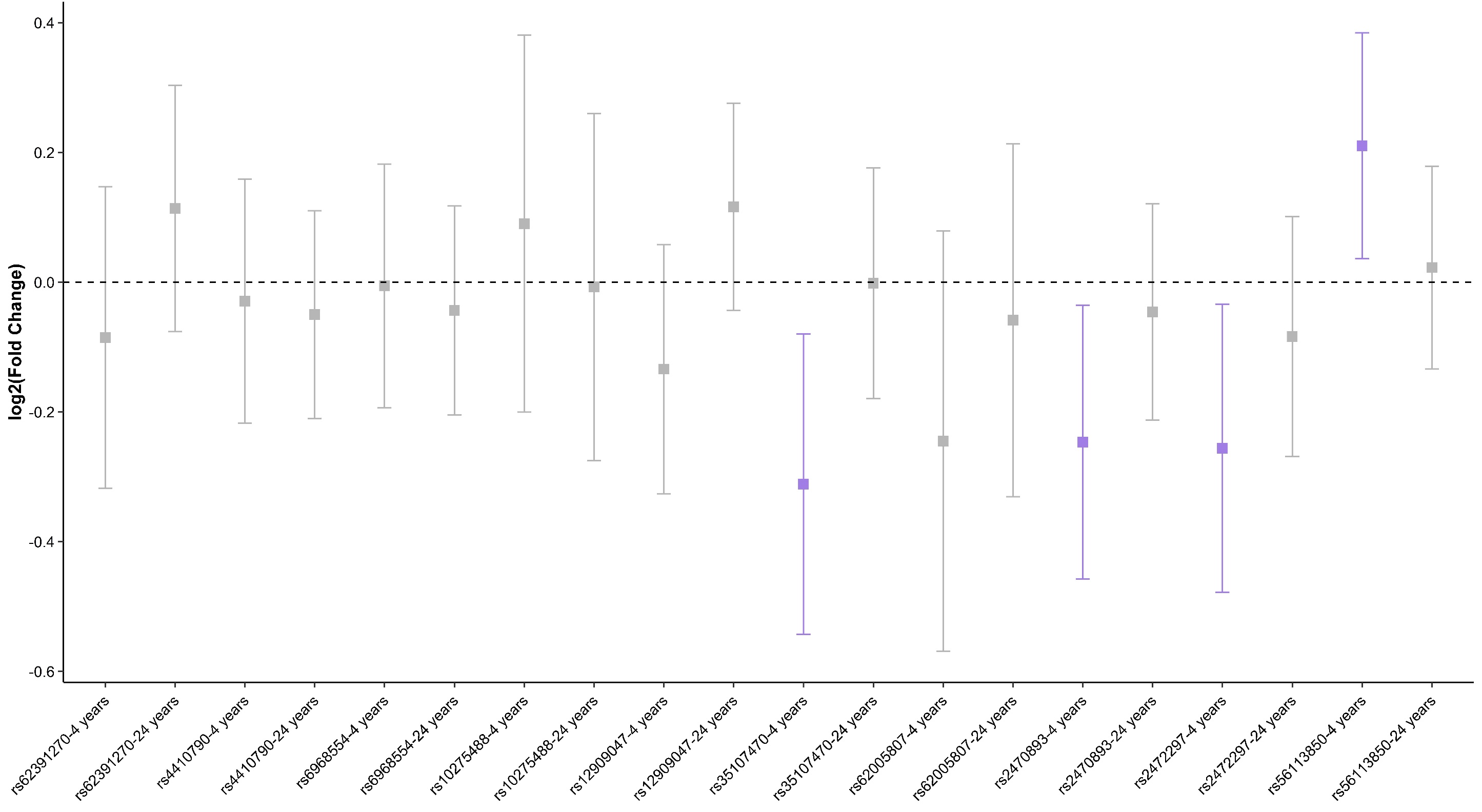


**Figure H.3. Association results of caffeine-metabolism associated genetic variants with 1,3-Dimethyluric acid in children and young adults.** Plot comparing the results of the exponential regression model evaluating the association of each SNP with metabolites measured in urine at 4 and 24 years of age. Analyses were carried out in both genotyping Waves of BAMSE (n= 492). The effect size of the association of each SNP (*x*-axis) with the metabolite levels is shown in terms of log_2_(Fold change) (*y*-axis) by boxes. Purple and orange boxes show the association effect estimate with metabolite levels measured in urine samples collected at the 4-year follow-up and 24-year follow-up, respectively (significant associations (p<0.05). The gray boxes represent non-significant results (p>0.05).


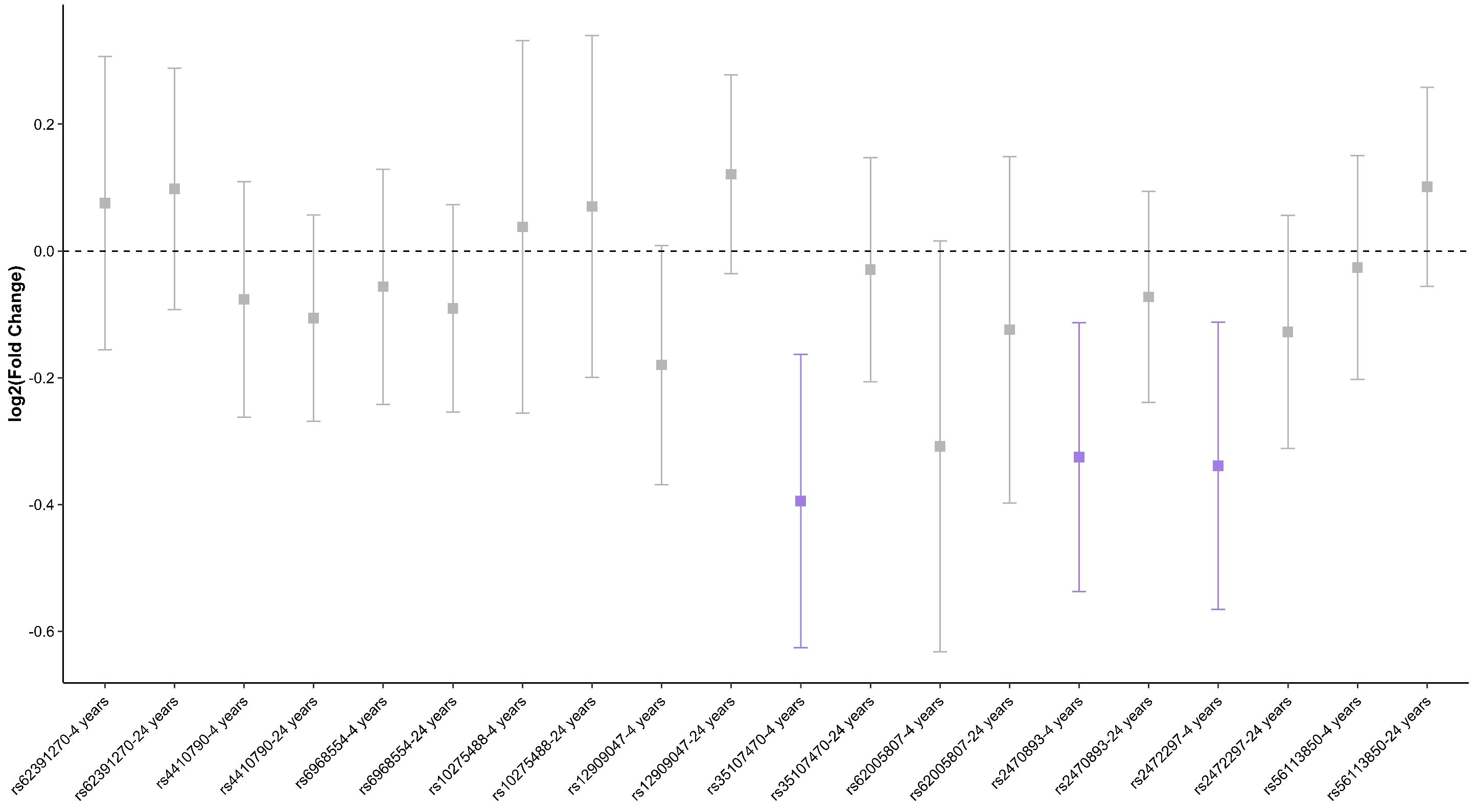


**Figure H.4. Association results of caffeine-metabolism associated genetic variants with (1,7)-Dimethyluric acid in children and young adults.** Plot comparing the results of the exponential regression model evaluating the association of each SNP with metabolites measured in urine at 4 and 24 years of age. Analyses were carried out in both genotyping Waves of BAMSE (n= 492). The effect size of the association of each SNP (*x*-axis) with the metabolite levels is shown in terms of log_2_(Fold change) (*y*-axis) by boxes. Purple and orange boxes show the association effect estimate with metabolite levels measured in urine samples collected at the 4-year follow-up and 24-year follow-up, respectively (significant associations (p<0.05). The gray boxes represent non-significant results (p>0.05).


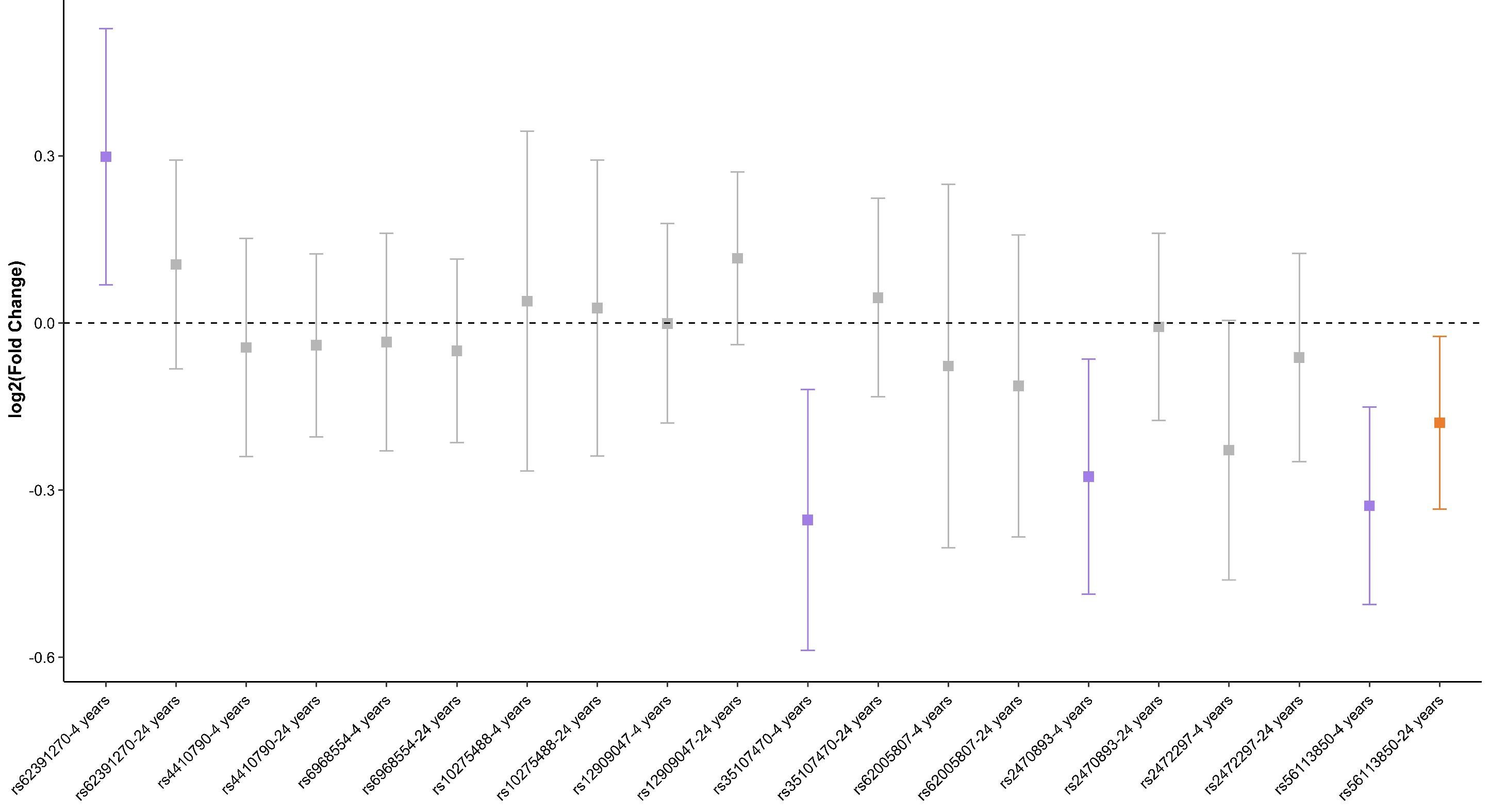


**Figure H.5. Association results of caffeine-metabolism associated genetic variants with 1,7-Dimethylxanthine in children and young adults.** Plot comparing the results of the exponential regression model evaluating the association of each SNP with metabolites measured in urine at 4 and 24 years of age. Analyses were carried out in both genotyping Waves of BAMSE (n= 492). The effect size of the association of each SNP (*x*-axis) with the metabolite levels is shown in terms of log_2_(Fold change) (*y*-axis) by boxes. Purple and orange boxes show the association effect estimate with metabolite levels measured in urine samples collected at the 4-year follow-up and 24-year follow-up, respectively (significant associations (p<0.05). The gray boxes represent non-significant results (p>0.05).


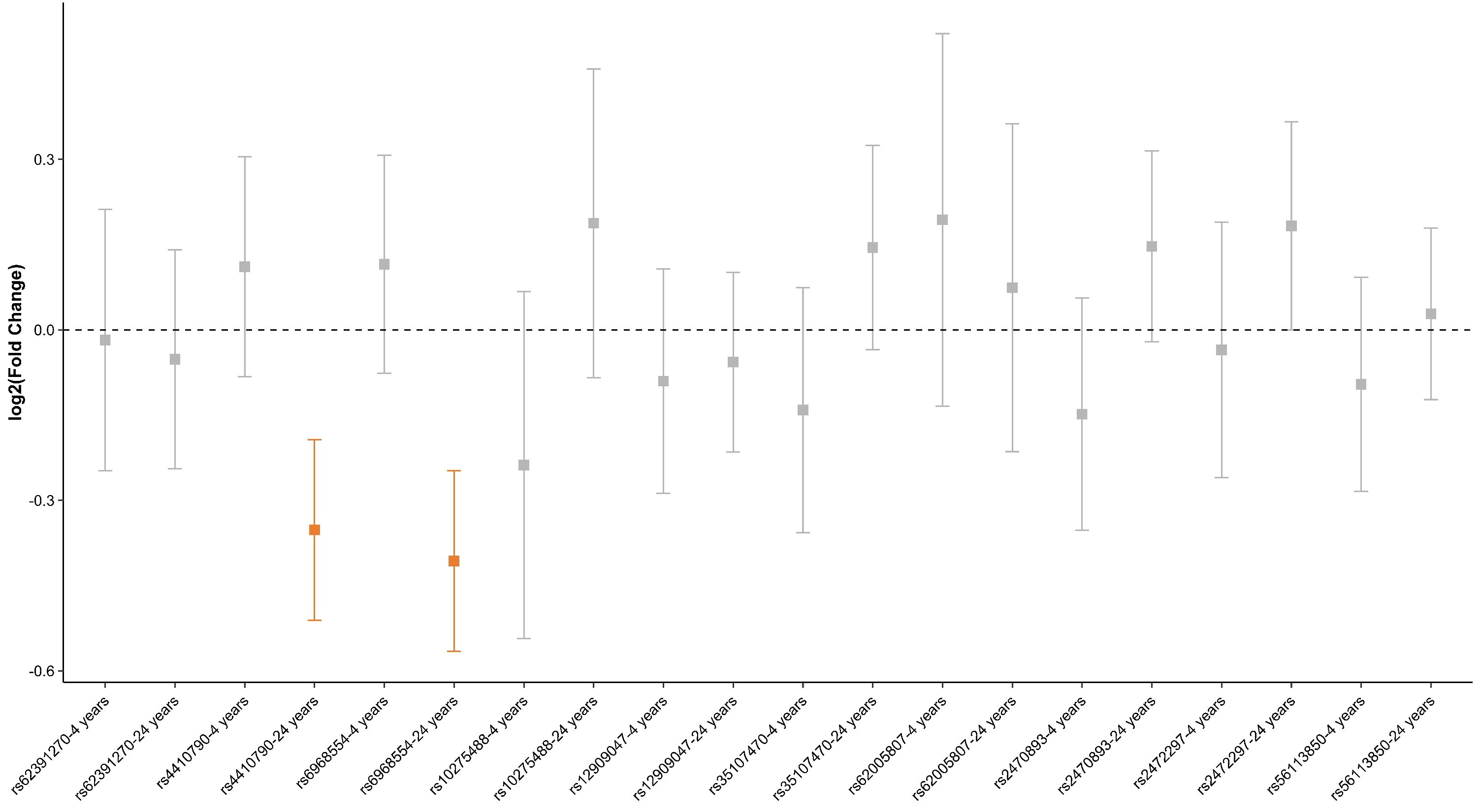


**Figure H.6. Association results of caffeine-metabolism associated genetic variants with 4-Hydroxycoumarin in children and young adults.** Plot comparing the results of the exponential regression model evaluating the association of each SNP with metabolites measured in urine at 4 and 24 years of age. Analyses were carried out in both genotyping Waves of BAMSE (n= 492). The effect size of the association of each SNP (*x*-axis) with the metabolite levels is shown in terms of log_2_(Fold change) (*y*-axis) by boxes. Purple and orange boxes show the association effect estimate with metabolite levels measured in urine samples collected at the 4-year follow-up and 24-year follow-up, respectively (significant associations (p<0.05). The gray boxes represent non-significant results (p>0.05).


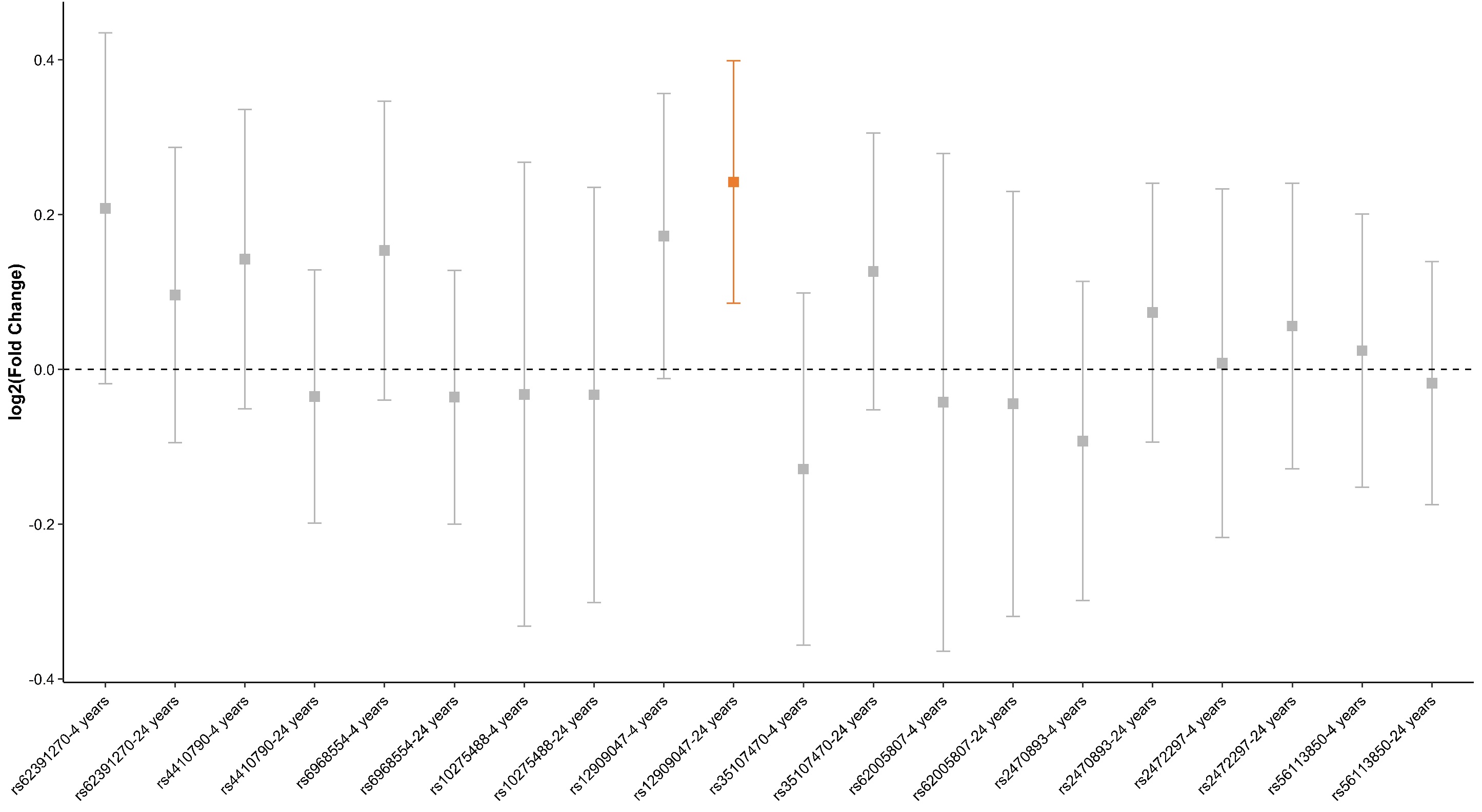


**Figure H.7. Association results of caffeine-metabolism associated genetic variants with Methyluric acid in children and young adults.** Plot comparing the results of the exponential regression model evaluating the association of each SNP with metabolites measured in urine at 4 and 24 years of age. Analyses were carried out in both genotyping Waves of BAMSE (n= 492). The effect size of the association of each SNP (*x*-axis) with the metabolite levels is shown in terms of log_2_(Fold change) (*y*-axis) by boxes. Purple and orange boxes show the association effect estimate with metabolite levels measured in urine samples collected at the 4-year follow-up and 24-year follow-up, respectively (significant associations (p<0.05). The gray boxes represent non-significant results (p>0.05).


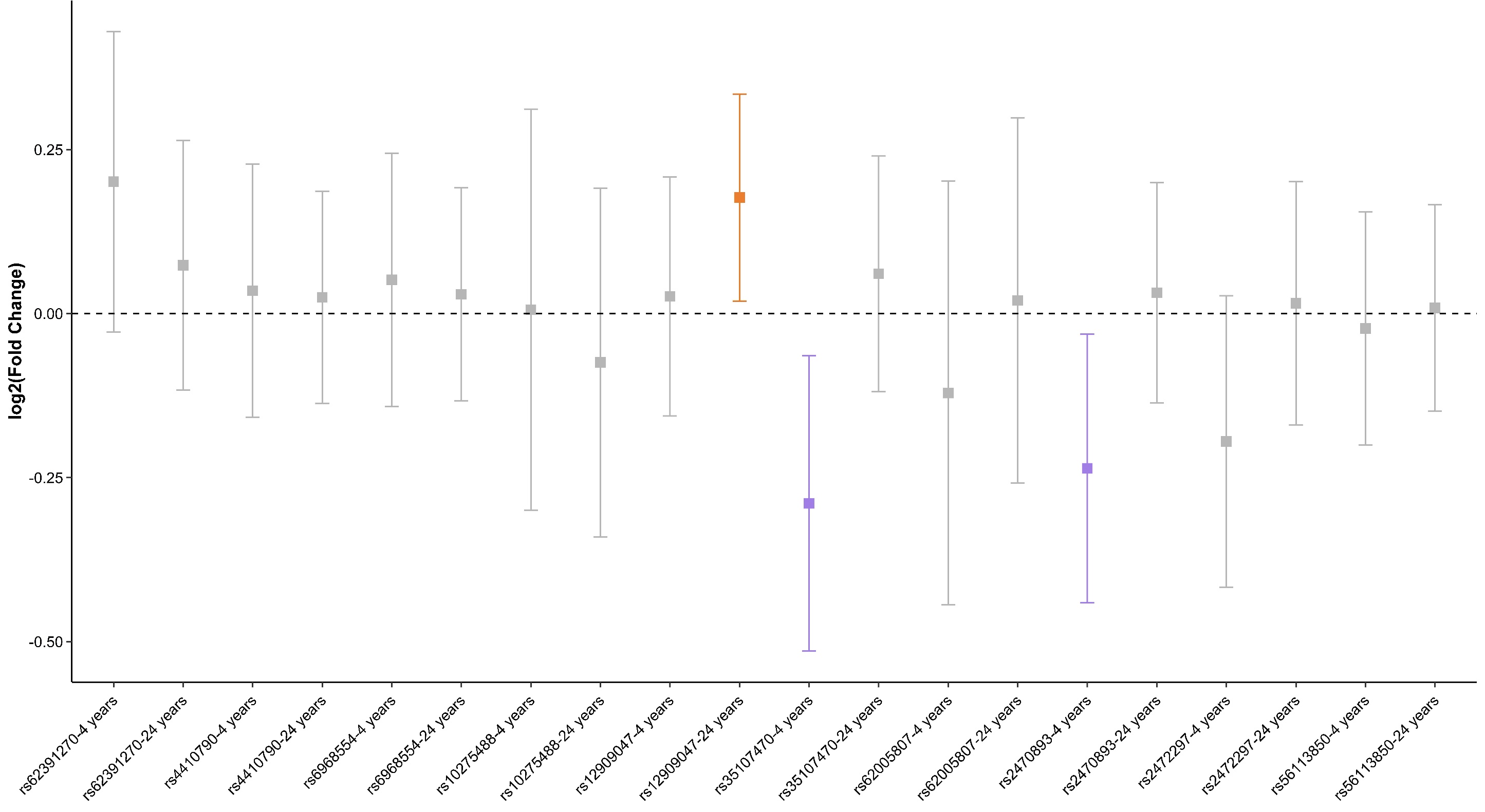


**Figure H.8. Association results of caffeine-metabolism associated genetic variants with 1-Methylxanthine in children and young adults.** Plot comparing the results of the exponential regression model evaluating the association of each SNP with metabolites measured in urine at 4 and 24 years of age. Analyses were carried out in both genotyping Waves of BAMSE (n= 492). The effect size of the association of each SNP (*x*-axis) with the metabolite levels is shown in terms of log_2_(Fold change) (*y*-axis) by boxes. Purple and orange boxes show the association effect estimate with metabolite levels measured in urine samples collected at the 4-year follow-up and 24-year follow-up, respectively (significant associations (p<0.05). The gray boxes represent non-significant results (p>0.05).


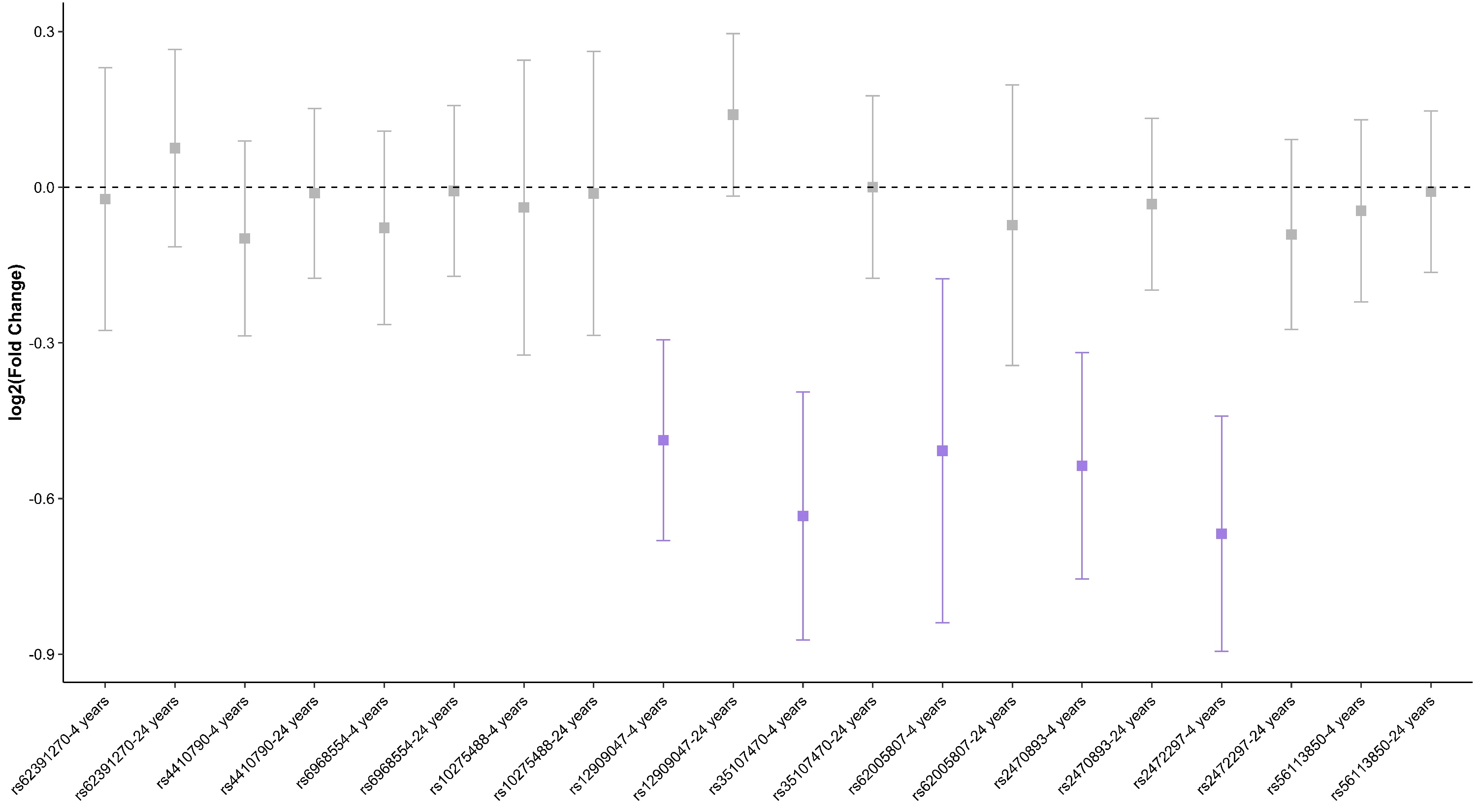
**Figure H.9. Association results of caffeine-metabolism associated genetic variants with 1,3,7-Trimethyluric acid in children and young adults.** Plot comparing the results of the exponential regression model evaluating the association of each SNP with metabolites measured in urine at 4 and 24 years of age. Analyses were carried out in both genotyping Waves of BAMSE (n= 492). The effect size of the association of each SNP (*x*-axis) with the metabolite levels is shown in terms of log_2_(Fold change) (*y*-axis) by boxes. Purple and orange boxes show the association effect estimate with metabolite levels measured in urine samples collected at the 4-year follow-up and 24-year follow-up, respectively (significant associations (p<0.05). The gray boxes represent non-significant results (p>0.05).


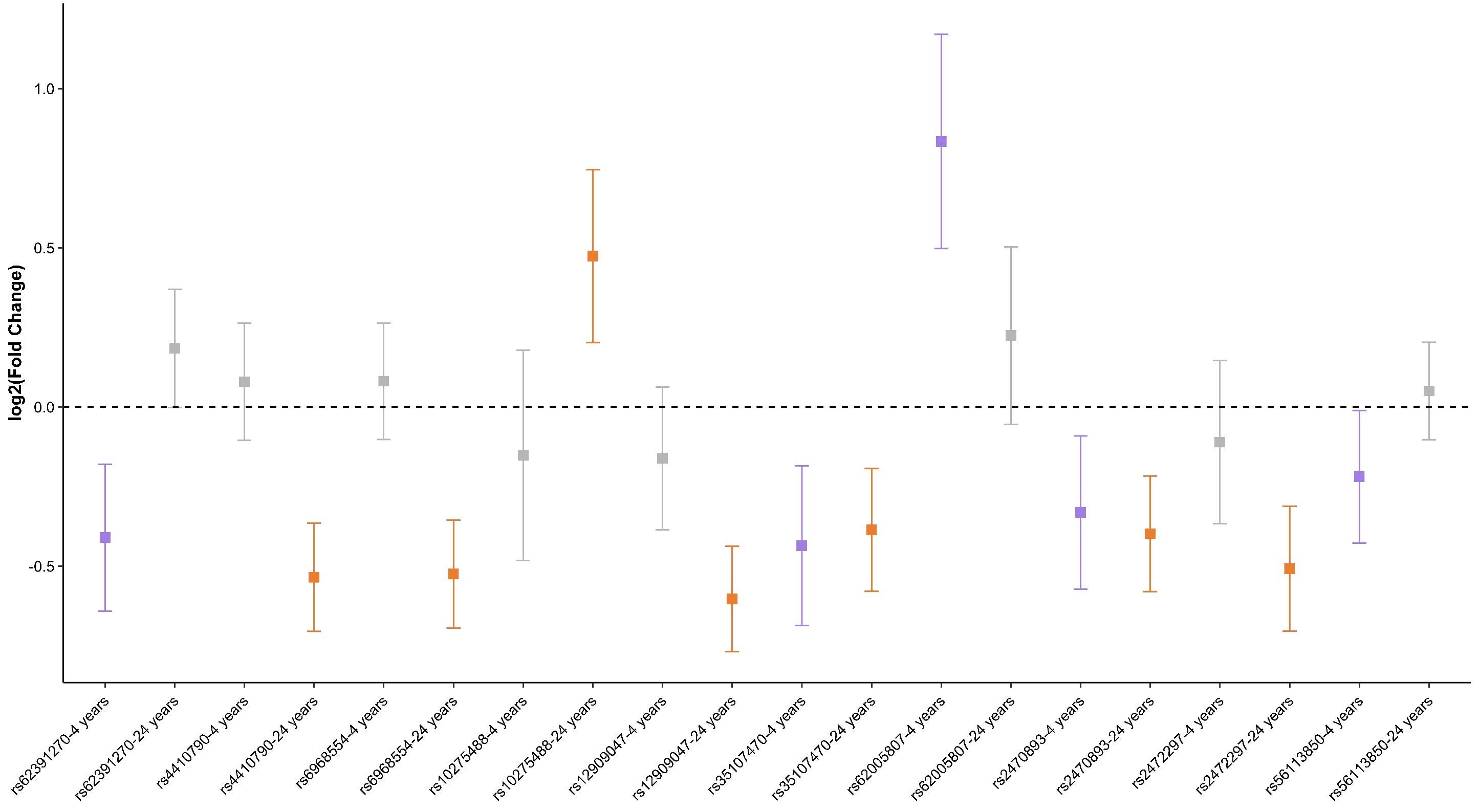


**Figure H.10. Association results of caffeine-metabolism associated genetic variants with Umbelliferone in children and young adults.** Plot comparing the results of the exponential regression model evaluating the association of each SNP with metabolites measured in urine at 4 and 24 years of age. Analyses were carried out in both genotyping Waves of BAMSE (n= 492). The effect size of the association of each SNP (*x*-axis) with the metabolite levels is shown in terms of log_2_(Fold change) (*y*-axis) by boxes. Purple and orange boxes show the association effect estimate with metabolite levels measured in urine samples collected at the 4-year follow-up and 24-year follow-up, respectively (significant associations (p<0.05). The gray boxes represent non-significant results (p>0.05).





**Figure H.11. Interaction between PM_10_ exposure during the prior year to biosampling and caffeine metabolisms-related SNPs, in relation to umbelliferone at 4 years of age.** Association analyses were performed in 473 children with available metabolite levels, air pollution exposure, and genome-wide genotype data. Log2 scale of the estimated fold change and the estimated 95% CI are shown on the y-axis for the SNP, air pollutant, and interaction terms (x-axis).

**
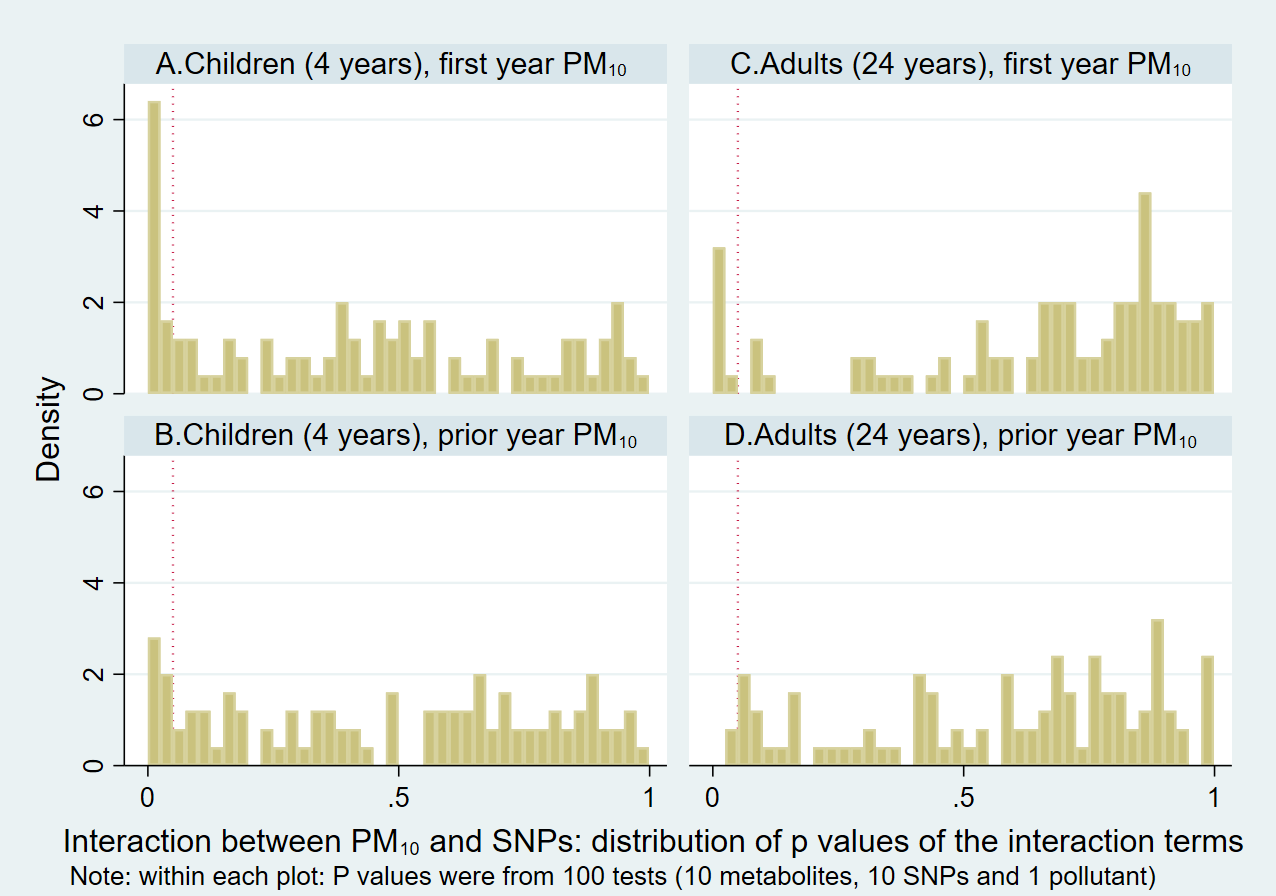
**

**Figure H.12. Distribution of p-value of the interaction between genetic variants and exposure to PM_10_ on urine metabolites in children and young adults.** The red dashed line indicates the nominal significance level (p=0.05). Panels A-B represent the results of the evaluation of the interaction on metabolites measured at 4 years of age. The results of metabolites measured during the 24-year follow-up are shown in panels C-D. A total of 682 and 452 young adults were included in the assessment of the interaction effect of genetic variants with first-year and prior-year air pollutants, respectively, on 24-year metabolite levels.

*In Figures H.12A and H.12B, the peaks near the nominal significance level (p=0.05) suggest the presence of significant interactions in children. Each distribution was found to be significantly different from a uniform distribution by a significant Kolmogorov-Smirnov test (p<0.001). Figures H.12C and H.12D show that the identified interactions in adults were more prone to type I errors (false positives). These results suggest that potential interactions between genetic variants related to caffeine metabolism and air pollution on caffeine and coumarin-derived metabolites are more robust in children.*
